# Liquid submerged fermentation by selected microbial strains for onion skins valorization and its effects on polyphenols

**DOI:** 10.1007/s11274-023-03708-y

**Published:** 2023-07-26

**Authors:** Francesca Anna Ramires, Anna Rita Bavaro, Isabella D’Antuono, Vito Linsalata, Leone D’Amico, Federico Baruzzi, Loris Pinto, Annamaria Tarantini, Antonella Garbetta, Angela Cardinali, Gianluca Bleve

**Affiliations:** 1 Lecce Unit, National Research Council, - Institute of Sciences of Food Procuction (CNR-ISPA), Lecce, 73100 Italy; 2 National Research Council, - Institute of Sciences of Food Procuction (CNR-ISPA), Bari, 70126 Italy; 3 University of Bari Aldo Moro, Plant and Food Science Department (Di.S.S.P.A), Soil, Bari, 70126 Italy

**Keywords:** Food industry by-product, Fermentation strategies, Biorefinery, Bioactive compounds recovery

## Abstract

Onion skins, actually recycled as organic fertilizers, could be used as a substrate in environmental-friendly bioprocesses to recover high-value bioactive compounds and food ingredients.

In this work, a bioprospecting method was carried out including 94 bacterial and 45 yeast strains from several agri-food and environmental niches to verify their ability to grow on onion skins as unique nutrients source.

Red and yellow onion skins were assessed by newly selected starter-driven liquid submerged fermentation assays mainly aimed at the release and modification of polyphenols through microbial activities. Fermented onion skins were also investigated as a inexpensive favourable source of microbial enzymes (amylases, proteases, lipases, esterases, cellulases, xylanases).

In red onion skins, the treatment with *Lactiplantibacillus plantarum* TB 11–32 produced a slight increase of bioactive compounds in terms of total phenolics, whereas with the yeast strain *Zygosaccharomyces mrakii* CL 30 − 29 the quercetin aglycone content increased of about 25% of the initial raw material.

In yellow onion skins inoculated, the highest content of phenolic compounds was detected with the yeast strain *Saccharomyces cerevisiae* En SC, while quercetin aglycone increased of about 60% of the initial raw material in presence of the bacterial strain *L. plantarum* C 180 − 34.

In conclusion, the proposed microbial pre-treatment method can be a potential strategy to re-use onion skins as a fermentation substrate, and as a first step in the development of a biorefinery process to produce value-added products from onion by-products.

**Supplementary Information:**

The online version contains supplementary material available at 10.1007/s11274-023-03708-y.

## Introduction

The world production of onion (*Allium cepa* L.) is about 47 million tons every year and, during the past 20 years, this horticultural crop became the second most important one (FAOSTAT 2018). Before harvest, onion bulbs are generally pulled from the soil to stop growth. Then, they are cured for a few days in the field to reduce excess moisture from the outer skin and neck to prevent excessive shrinkage of the onion. During storage this step allows also for color development. A huge amount of waste (stalk, roots and skins) derives from industrial processing of onions. In Europe, a by-product production of about 50,000 tons per year was registered by Spain, Netherlands, and the UK, the main European onion producers (Roldan et al., 2008; Sharma et al. 2016; Santiago et al. 2020).

The onion wastes are rich in fiber and bioactive compounds and represent a source to develop different bioproducts such as polyphenols, pigments, organic acids, enzymes, and bioenergy (Sagar and Pareek 2020). However, the different chemical composition of onion waste categories limits their use as whole waste.

Onion skins removed from edible bulbs represent the major waste of onion processing, since they are unsuitable as landfill disposal or animal feed because of their composition and smell. Therefore, sustainable onion production requires the valorization of their skins by turning this waste into “by-product” (Gontard et al. 2018). Onion skin waste is a source of phenolic chemicals, primarily quercetin and its derivatives, luteolin, and kaempferol, myricetin, isorhamnetin derivatives and carbohydrates (Benito-Román et al. 2020; Sagar et al. 2020). Onion skin cell walls show a very complex structure, mainly formed by pectin (42.4%), hemicelluloses (36.6%), and cellulose (21%) (Munir et al. 2018). This by-product is also an interesting source of pectin, which is highly requested at global level (Kim et al. 2019) and the phenolic compounds can have many pharmacological properties, such as antioxidant, antimicrobial, anticarcinogenic, antimutagenic activities, and their antidiabetic potential (Benitez et al., 2011; Ren and Zhou 2021).

The theory of waste valorization is strictly associated with sustainable technologies for the recycling and the reuse. Fermentation is one of the most promising frontiers to start from biomass feedstock and, through various techniques such as solid-state fermentation (SSF), submerged fermentation (SmF), or anaerobic digestion to recover the nutrients by a biological extraction process, by creating value-added products and “green” energy (Carmona-Cabello et al. 2018).

Whole microbial cells can act on the cell wall matrix, leading to the bioconversion of the plant fibers i.e. cellulose, hemicellulose, pectin and lignin, and allowing the release of polyphenols and organic acids (Barcelos et al. 2020; Ramires et al. 2020; Sayago-Ayerdi et al., 2021). Several studies demonstrated that during fermentation of agri-food wastes microbial hydrolitic enzymes, such as amylase, protease, β-glucosidase, xylanase, cellulase can break down cell wall components and consequently facilitate phenolic mobilization, whereas esterase and lipase can be responsible of phenolics evolution essentially by esterification (Bei et al. 2018; Dey et al. 2016; Hur et al. 2014; Yin et al. 2020). The predominance of one or more of these enzymatic activities, positively correlated with the increase of the release or the bioavailability of phenolic compounds, can be considered a key attribute for the selection of new candidate microbial starter for the treatment of agri-food by-products.

The fermentation process has been considered as an efficient method to obtain the chemical modification of phenolic compounds from agro-industral by-products, and to enhance their release and eventually to increase the antioxidant activity (Gulsunoglu-Konuskan and Kilic-Akyilmaz 2022). Improvement of bioactive compounds extraction from plant products using a microbial fermentation process has been reported before, e.g., for apple pomace, black soybeans and Larrea tridentate leaves olive mill wastewater, orange skin waste (Cheng et al. 2013; Ramires et al. 2020). In SmF, the microorganisms develop in a liquid medium obtaining the nutrients required for their growth and metabolic activities by the raw material. This process allows the monitoring of the relevant parameters for microbial growth, such as pH and temperature, with very high efficiency.

Several fermentation approaches were explored for potential re-using onion wastes by lignocellulosic biomass treatment, such as for bioethanol production in presence (Kim et al. 2019) or not (Ganguly et al. 2021) of a enzymatic saccharification step for bio-sugar production and for producing acetone, butanol, ethanol using *Clostridium* culture (Poe et al. 2020). Also, acetic acid was obtained by fermenting dried onion waste with *Acetobacter acetii* (Kim et al. 2019).

In this study, two different onion cultivars, the red onion cv “*Cipolla rossa di Tropea*” and the yellow onion cv *Recas* were chosen, since they are common in commercial productions in Italy and Spain, respectively. The traditional and commercially relevant Italian Protected Geographic Indication (PGI) onion variety “*Rossa di Tropea*” from Calabria Region (Italy) is an outstanding food product with several peculiar organoleptic properties and a distinctive taste made by a delicate and persisting aroma. The yellow cv *Recas* is late cycle and long-day onion, with a good firmness and high density and good storage capacity. Benitez et al. (2011) reported that the skins, which are rich in polyphenols and flavonoids and a relative high antioxidant activity, could be considered as a potential functional ingredient. Moreover, the remarkable bioactivity of red onion “*Cipolla rossa di Tropea*” skins suggested several potential applications in different industrial sectors as nutraceuticals, pharmaceuticals and food (Celano et al. 2021).

The main objective of this study is to valorize onion skins by using a microbiological treatment able to improve the release and the extraction of polyphenols content of these by-products.

In this study microorganisms (yeasts and bacteria) from different natural origins were selected for their ability to grow in presence of red and yellow onion skins as unique nutrient sources.

SmF approach on red and yellow onion skins, inoculated with selected microbial starters, was applied to enhance the release of polyphenols as well as on changes in their profile and relative amount of single polyphenols. By this approach, an improved recovery of polyphenols from the onion skins and an enrichment in the aglyconic form of specific phenolic compounds, are expected. The hypothesis is that microorganisms can (i) act by hydrolytic enzymes on the vegetable cell wall structure and glycosidic linkages, releasing polyphenols and also (ii) use their secondary metabolism to promote the chemical modification of phenolic compounds (Slama et al. 2021; Gulsunoglu-Konuskan et al. 2021). Following this route, the major microbial enzymatic activities found in the fermented products were also investigated.

## Materials and methods

### Feedstock sampling

Red onion skins (cv *Cipolla rossa* di *Tropea*) were kindly supplied by the Italian company Azienda Agricola Veltri srl (CZ, Italy), yellow onion skin samples (cv *Recas* variety Citation) were provided by PROCECAM (Association of Onion Producers of Castilla-La Mancha, Spain). Onion skin samples were packed in aerated bags at production plant sites in June 2021 and transferred to the laboratory in 3–5 days, then samples were immediately grinded by a laboratory knife mill (Grindomix GM 200, RETSCH Gmbh, Haan, Germany) at maximum speed for 5 min and stored at – 20 °C for further experiments.

### Microbial species and culture conditions

In this study, a number of 94 bacterial strains were chosen among two phyla frequently associated with plant material and the production of fermented foods:

- Proteobacteria (alphaproteobacteria *Acetobacter, Agrobacterium, Sphingomonas*; gammaproteobacteria *Dickeya, Enterobacter, Erwinia, Hafnia, Pantoea, Pectobacterium, Pseudomonas, Serratia*);
- Bacillota (synonym Firmicutes, Oren and Garrity 2021) (*Bacillus, Lactiplantibacillus, Leuconostoc, Levilactobacillus, Paenibacillus, Pediococcus, Staphylococcus*).

Also, a number of 45 yeast strains were selected among species (*Aureobasidium, Candida, Debaryomyces, Geotrichum, Hanseniaspora, Kluyveromyces, Metschnikowia, Pichia, Rhodotorula, Saccharomyces, Trichosporon, Zygosaccharomyces*) generally associated to food fermentation and treatment of waste/by-products of vegetable origin.

The entire collection set of yeast and bacterial strains were selected from the CNR-ISPA microbial collection (Table S1 and S2).

*Bacillus* spp. strains were grown on Nutrient agar medium (Merck KGaA, Darmstadt, Germany) at 25 °C for 2–3 days; *Bacillus simplex* strains, *Paenibacillus* spp., *Sphingomonas* spp. were grown on Tryptic soy agar (Merck KGaA, Darmstadt, Germany) at 30 °C for 2–3 days; LAB strains were grown on Man, Rogosa and Sharpe (MRS) agar medium (Microbiol Diagnostics, Cagliari, Italy) at 30 °C for 3–5 days under anaerobic conditions; *Staphylococcus* spp. were grown on MRS Agar with replacement of glucose as carbon source with sucrose and addition of 7.5% marine salts (VibrantSea, Seachem Laboratories Inc., Madison GA, USA) at 37 °C for 3–5 days, under anaerobic conditions. *Pseudomonas* and *Enterobacteriaceae* strains were grown on Nutrient Broth added with 1% (w/v) glucose at 30 °C for 1–2 days. *Acetobacter* spp. were grown on GYC medium (Hommel 2014) modified as follows (mGYC): 50 g/L glucose (VWR Chemicals, Leuven, Belgium), 10 g/L yeast extract (Merck KGaA, Darmstadt, Germany), 2 g/L CaCO_3_ (Merck KGaA, Darmstadt, Germany) for 48–72 h at 28 °C. All media were added with 0.05 g/L nystatin.

Yeast strains were all grown on Sabouraud dextrose agar medium (LABM, Heywood, Lancashire, UK) added with 0.1 g/L of ampicillin (Sigma-Aldrich, Darmstadt, Germany) and 0.05 g/L of kanamycin (Sigma-Aldrich, Darmstadt, Germany) and incubated at 30 °C for 2–4 days.

### Yeast and bacteria collection screening on onion skins-based media

Microbial media for yeast and bacteria collection screening were prepared following the indications of Niu et al. (2017) with some modifications: 2 g/L casein peptone (Sigma-Aldrich, Darmstadt, Germany), 2 g/L soy peptone (Sigma-Aldrich, Darmstadt, Germany), 2 g/L NaCl (Sigma-Aldrich, Darmstadt, Germany), 20 g/L agar (Sigma-Aldrich, Darmstadt, Germany) and a specific percentage (w/v) of ground onion skins. Preliminary experiments have been performed in order to evaluate the suitable quantity of the onion skins to prepare the agar media (data not shown). The final selected percentages of the ground onion skins added to the mixture were 3-4-5% (w/v). These concentrations are corresponding to a final hydrated ground onion skins preparation percentage of 15-20-25% (w/v). The weight increase of hydrated onion skins was measured after the incubation of the initial samples in presence of water excess for 30 min and the draining until the weight was stable. It resulted about 5-fold higher than the initial weight value.

The incubation conditions were the same used for the growth on laboratory media. Microbial strains were screened for their ability to survive and grow on different onion skins (OS) concentrations by applying an arbitrary scale: 0, no growth; 1, slight growth; 2, medium growth; 3, intense growth (similar to the growth optimal medium, used as control); 4, > growth on the optimal medium.

The growth scores were used to obtain a Weighted Mean Growth (WMG) value for each strain. The equation was defined as follows:

WMG = [(N1 × 2) + (N2 × 3) + (N3 × 5)]/10.

N1 = scale growth on 15% concentration OS.

N2 = scale growth on 20% concentration OS.

N3 = scale growth on 25% concentration OS.

2 = multiplier value for the growth at 15% concentration OS.

3 = multiplier value for the growth at 20% concentration OS.

5 = multiplier value for the growth at 25% concentration OS.

10 = numerator (sum of the multiplier values).

### Laboratory-scale fermentation assays

A submerged batch fermentation was set up for feedstocks treatment. The raw material was homogenized and used to prepare samples to be singularly inoculated with selected microbial strains. The final volume fermentation in glass jars (100 mL) was prepared adding 25 g of hydrated onion skins and 50 mL drinking water. Prepared mix was heat treated in water bath at 90 °C for 10 min, cooled and inoculated with 25 mL of fresh microbial suspension prepared as follows. Firstly, microbial inoculum was prepared growing the selected strains in specific media, incubating at 28–30 °C for 16–48 h under mild stirring. The final inoculum of 4 different starter microorganism for each onion skins source (Table 1) was adjusted in 25 mL starter solution (casein peptone 0.5 g/L; soy peptone 0.5 g/L; NaCl 2 g/L) to obtain a final value corresponding to 7 log_10_ CFU/ml for yeasts, and 8 log_10_ CFU/ml for bacteria and added to the heat-treated feedstock mixture. The fermentation was carried out for ten days at 30 °C under aerobic static conditions.

**Table 1:** Bacterial and yeasts strains selected by considering the highest Weighted Mean Growth (WMG) values obtained after the plate screening step in presence of increasing concentrations of red or yellow onion skins as unique nutrient source

*Red onion skins*		
**Microbial Species**	**WMG value**	**Reference Code**
*Acetobacter tropicalis* G25b	3.8	14
*Lactiplantibacillus plantarum* TB 11–32	2.3	30
*Metschnikowia pulcherrima* 2047	1.8	45
*Zygosaccharomyces mrakii* CL 30 − 29	2.8	57
***Yellow onion skins***		
**Microbial Species**	**WMG value**	**Reference Code**
*A. tropicalis* G25b	3.5	14
* L. plantarum* C 180 − 34	2	28
*Candida boidinii* A5y	3	33
*Saccharomyces cerevisiae* En SC	3.5	71

### Microbiological, physical-chemical analyses, enzyme activity assay of fermented onion skins

#### Microbiological assays

Microbiological analyses of samples were carried out following the procedure described by Ramires et al. (2022) with some modifications. Ground onion skins were incubated 1 g/L (*w*/*v*) peptone water (Sigma-Aldrich, Darmstadt, Germany) for 1 h under agitation (150 rpm) at room temperature; an aliquot of liquid fraction of heat treated onion skins and fermented samples were diluted with 1 g/L (*w*/*v*) peptone water and analyzed on agar media plates:

- Plate Count Agar (PCA, Heywood, Lancashire, UK) with 0.05 g/L nystatin (Sigma-Aldrich, Darmstadt, Germany) and incubated for 48–72 h at 30 °C was used for total bacterial count;
- Violet Red Bile Glucose Agar (VRBGA, LABM, Heywood, Lancashire, UK) incubated at 37 °C for 18–24 h, for Enterobacteriaceae;
- Violet Red Bile Agar (VRBA, LABM, Heywood, Lancashire, UK) incubated at 37 °C for 24–48 h, for coli–aerogenes bacteria; Baird Parker Agar Base (BP, LABM, Heywood, Lancashire, UK) incubated at 37 °C for 24–48 h, for Staphylococci;
- *Bacillus* ChromoSelect Agar (BCSA, Sigma-Aldrich, Darmstadt, Germany) with Polymyxin B supplement (Sigma-Aldrich, Darmstadt, Germany) incubated at 30 °C for 24–48 h, for *Bacillus* spp.;
- *Pseudomonas* agar (LABM, Heywood, Lancashire, UK) added with CFC supplement (LABM, Heywood, Lancashire, UK) incubated at 35 °C for 24–48 h, for *Pseudomonas* spp.;
- Sulphite-Polymyxin-Sulphadiazine Agar (SPS, Biolife Italiana srl, Milano, Italy) incubated at 35–37 °C for 18–48 h under anaerobic conditions, for the detection of *Clostridium* spp.;
- *Acetobacter* spp. were grown for 48–72 h at 28 °C on modified GYC medium (mGYC) containing 50 g/L glucose (VWR Chemicals, Leuven, Belgium), 10 g/L yeast extract (Merck KGaA, Darmstadt, Germany), 2 g/L CaCO_3_ (Sigma-Aldrich, Darmstadt, Germany) (Hommel 2014) and 0.05 g/L nystatin (Sigma-Aldrich, Darmstadt, Germany).
- Yeast total count was determined on Sabouraud dextrose agar medium (LABM, Heywood, Lancashire, UK) added with 0.1 g/L of ampicillin (Sigma-Aldrich, Darmstadt, Germany) and 0.05 g/L of kanamycin (Sigma-Aldrich, Darmstadt, Germany) at 30 °C for 3–4 days.

### pH and liquid/solid ratio

Fermented and non-fermented samples were filtered by polyamide filter 355/51 (Saati, Milan Italy) using a vacuum pump in order to separate solid and liquid portions. The solid and liquid fractions for each sample were measured by weighing and the results have been expressed as their ratio. The pH was measured by the pHmeter (Hanna Instruments srl, Ronchi di Villafranca Padovana, Italia).

### Enzymatic activity assays

Enzyme activity assays for α-amylase, protease, esterase, lipase, cellulase and endo-xylanase were carried out to evaluate the amount of these enzymes deriving from both microorganisms and plant cells in the liquid portion of the samples. All experiments were conducted in triplicate.

For crude enzyme solution preparation, an aliquot of each fermented and unfermented sample was filtered by polyamide filter 355/51 (Saati, Milan, Italy), and the resulting liquid fraction centrifuged at 15,000 x *g* for 15 min at 4 °C and the resulting supernatant was used for the assays.

### α-Amylase activity assay

The α-amylase assay was performed as described by Ramires et al. (2022). Briefly, the reaction mixture consisted of 50 µL substrate solution (1% potato starch in pH 7 phosphate buffer), 93 µL of phosphate buffer at pH 7 and 72 µL of raw enzyme solution. One Unit (U) of activity of α-amylase is defined as the amount of enzyme required to release one micromole of glucose reducing-sugar equivalents per minute.

### Protease activity assay

The method by Walter (1984) and Moyano et al. (1996), modified with the use of casein (0.66% (w/v) in 50 mM Tris-HCl buffer pH 8 as a substrate, as proposed by Sigma-Aldrich Company, was followed for this activity test. Protease enzyme activity was measured as a change in absorbance at 765 nm using the microplate reader Infinite M200 PRO (Tecan, Switzerland). One unit of enzyme activity was expressed as 1 µmol of tyrosine min^− 1^ mg protein^− 1^.

### Esterase activity assay

The carboxyl ester hydrolase (esterase) activity was determined using a modified spectrometric method (Lopes et al. 2011) following the hydrolysis of *p*-nitrophenylbutyrate (p-NPB) to *p*-nitrophenol at 37 °C for 5 min. One unit (U) of esterase activity was defined as the amount of esterase needed to release 1 µmol of p-nitrophenol per minute from p-NPB.

### Lipase activity assay

A spectrometric method was also performed for the measurement of lipase activity using p-nitrophenyl palmitate (p-NPP) as a substrate (Park et al. 2007). The assay was performed on 1 mL of crude enzyme solution properly diluted as described by Maiorano et al. (2022). One unit (U) of lipase activity was defined as the amount of lipase required to release 1 µmol of *p*-nitrophenol from *p*-NPP in 1 min under the corresponding conditions.

### Endo-xylanase activity assay

This assay specifically detects the activity of endo-xylanase and not the activity of the enzyme xylosidase or exo-xylanase. The endo-xylanase activity of the supernatants was assayed using Xylanase Assay kits (XylX6 method) (Megazyme, Bray, Ireland), by following the manufacturer’s instructions as described by Maiorano et al. (2022). The endo-xylanase activity (one unit) was defined as 1 µmol of pNP released from XylX6 per minute.

### Cellulase activity assay

The catalytic activity of microbial cellulases was tested using a cellulase assay kit (CellG5, Megazyme, Bray, Ireland), as the method provided by the manufacturer, and as described by Maiorano et al. (2022). The cellulase activity (one unit) was defined as 1 µmol of pNP released from CellG5 per minute.

### Phenolics content analyses

#### Polyphenols extraction

Fermented samples were lyophilized and about 0.50 g of each dried sample were mixed with 20 mL of 60% EtOH (ethanol/water 60:40) containing 0.5% v/v trifluoroacetic acid (TFA). The mixtures were kept under constant stirring (150 rpm) for 30 min at room temperature and the extracts were collected after centrifugation at 4500 x *g* for 10 min. The extractions were performed twice adding 10 mL of fresh solvent to the pellets and the supernatants were combined for further analysis.

### Determination of total phenol content

Total Phenol Content (TPC) in the extracts was determined by the Folin-Ciocalteu colorimetric method as reported by Cicco et al. (2009) with slight modifications. Briefly, 100 µL of each extract was mixed with 500 µL of Folin-Ciocalteu phenol reagent and 500 µL of distilled water. After agitation and 3 min standing 1 mL of 20% sodium carbonate (Na_2_CO_3_) and 2.9 mL of distilled water were added. Samples were left in the dark at 40 °C for 20 min and the absorbance was spectrophotometrically measured at 750 nm. The calibration curve was plotted versus concentrations of gallic acid ranging from 25 to 400 µg/mL, used as a standard. The results were expressed as mg of gallic acid equivalents (GAE) per gram of dry sample (DW). Two parallel determinations of each sample were performed and average values were calculated.

### HPLC-DAD analysis

Aliquots of hydroalcoholic extracts, filtered on 0.45 μm with CA filters, were analyzed by HPLC-DAD using the Agilent 1260 Infinity Series Chromatograph system, supplied with Agilent Open Lab CDS Chem Station Software (Palo Alto, CA, USA). The instrument was equipped with 1260 HIP Degasser, G1312B binary Pump, G1316A Thermostat and G4212B DAD Detector. For separation, analytical 5 μm Phenomenex Luna C18 (4.6 × 250 mm) column (Phenomenex Torrance, CA, USA) was used. The mobile phase was methanol (solvent A) and acetic acid/water (5:95 v/v) (solvent B) and the gradient profile was: 0–25 min, 15–40% A, 25–30 min, 40% A (isocratic), 30–45 min, 40–63% A, 45–47 min, 63% A (isocratic), 47–52 min, 63–100% A, 52–56 min, 100% A (isocratic), with a constant flow of 1 mL/min. Furthermore, the identification of the the main phenolic compounds was performed comparing the spectra and retention time of the pure available standards, as reported by D’Antuono et al. (2018).

### Statistical analysis

All data represent the mean of at least three independent replicates (n = 3). Data are presented as mean values ± standard error of the mean values. For microbiological analyses, t-test assay was applied to compare differences between microbial counts; for L/S ratio and enzymatic activities, Kruskal-Wallis test followed by Dunn’s multiple comparisons test were applied to establish significant differences among means values (*p* < 0.05). Statistical comparisons were performed using Prism 6 software (La Jolla, CA, USA, www.graphpad.com).

The mean values related to phenolic content were subjected to one-way ANOVA followed by Fisher’s post hoc test. Results analysis was performed using the software STATISTICA 6.0 (StatSoft, Tulsa, OK) and was considered as significantly for *p* ≤ 0.05. The OriginPro 2016 software (OriginLab, USA) was used for further analyses. Principal component analysis (PCA) was used to compare microbiological, chemical, biochemical parameters associated with the fermented and unfermented samples.

## Results and discussion

### Microbial screening and selection of best candidates for fermentation experiments

To guide the efforts to assess a simplified method for establishing best microbial strains able to use onion skins as the unique fermentation substrate and possibly improve the following extraction of bioactive compounds (polyphenols) from this by-product, a wide range of bacterial and yeast species were assayed. A total number of 94 different bacterial and of 45 yeast strains belonging to the CNR-ISPA collection were used for the screening on the two feedstocks (red and yellow onion skins).

As the first step toward the selection of functional microorganisms, a simple growth medium, prepared with different percentages of onion skins and a negligible quantity of additional nutrients, was set up to check the growth capabilities of different strains.

Microbial growth was measured by applying an arbitrary scale (as described in the Material and Methods section). A query database was elaborated reporting the growth scores of the entire microbial collection used in this study on the tested feedstocks.

In this study, the obtained growth scores were used to confer a Weighted Mean Growth (WMG) value to each strain. By this score the ability of each microbial strain to grow at increasing feedstock concentrations was pondered and encouraged. The WMG value was used to select the best-performing strains as candidates starters to drive the subsequent step of onion skins submerged fermentation experiments (Table S1 and S2).

The analysis of WMG for bacteria ranged from 0 to 3.8 with an average value of 0.7 for red onion skins, and from 0 to 3.5 with an average value of 0.4 for yellow onion skins. Among bacteria, a number of 37 and 68 strains did not grow on agar plate supplemented with at least the 15% hydrated red and yellow onion skins, respectively, and for this reason they were not considered anymore during the study. Only the *Acetobacter tropicalis* G25 b strain showed a score near 4 for red and yellow onion skins.

**Fig. 1:**
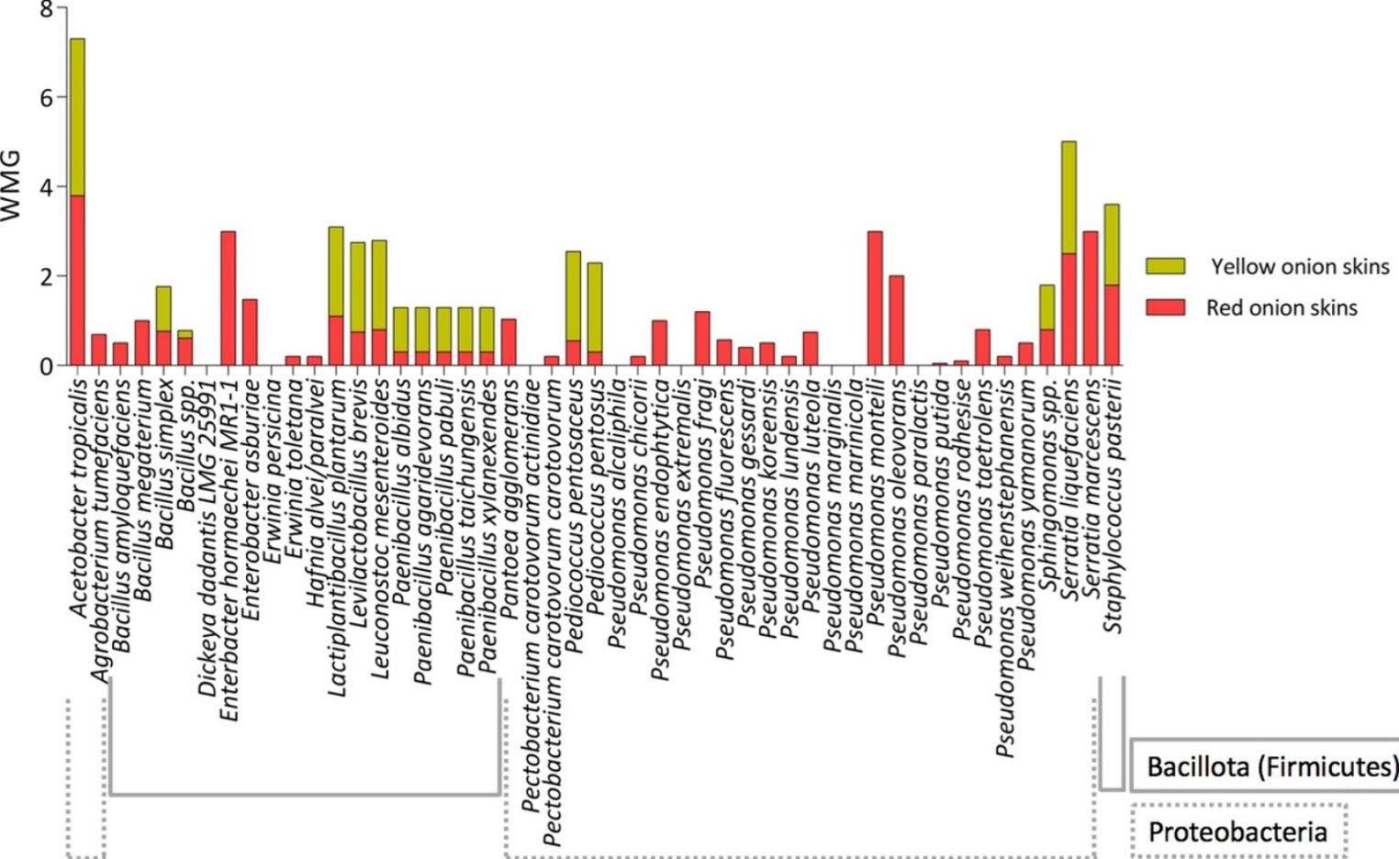
Bacterial isolates grouped in genera/species ability to grow in presence of different percentages of onion skins homogenate as main nutrient source in plate medium. The reported score is the average value of all the weighted mean growth (WMG) value for the strains belonging to each genus/species

Among Proteobacteria, the average WMG resulted 0.7 and 0.07 for red and yellow onion skins, respectively, whereas for Bacillota (syn. Firmicutes) these values were 0.7 and 1.05 (Table S1). These evidences revealed that tested members of Bacillota phylum were more able to adapt for growth in presence of these two peculiar feedstocks than strains belonging to Proteobacteria and that the two considered Phyla showed a broader range of sensitivity to yellow onion skins than to red ones. The ability to grow in presence of onion skins as main nutrient source varied among all bacteria genera (Fig. 1).

*Acetobacter tropicalis* strains were the best performers for both the two types of onion skin samples, followed by *Serratia liquefaciens*, *Staphylococcus pasteurii, Levilactobacillus brevis*, *Lactiplantibacillus plantarum*, *Leuconostoc mesenteroides*. *Pediococcus* spp., and finally *Paenibacillus* spp., whereas *Sphingomonas* and *Bacillus simplex* strains showed a preferential growth in presence of yellow onion skins.

**Fig. 2:**
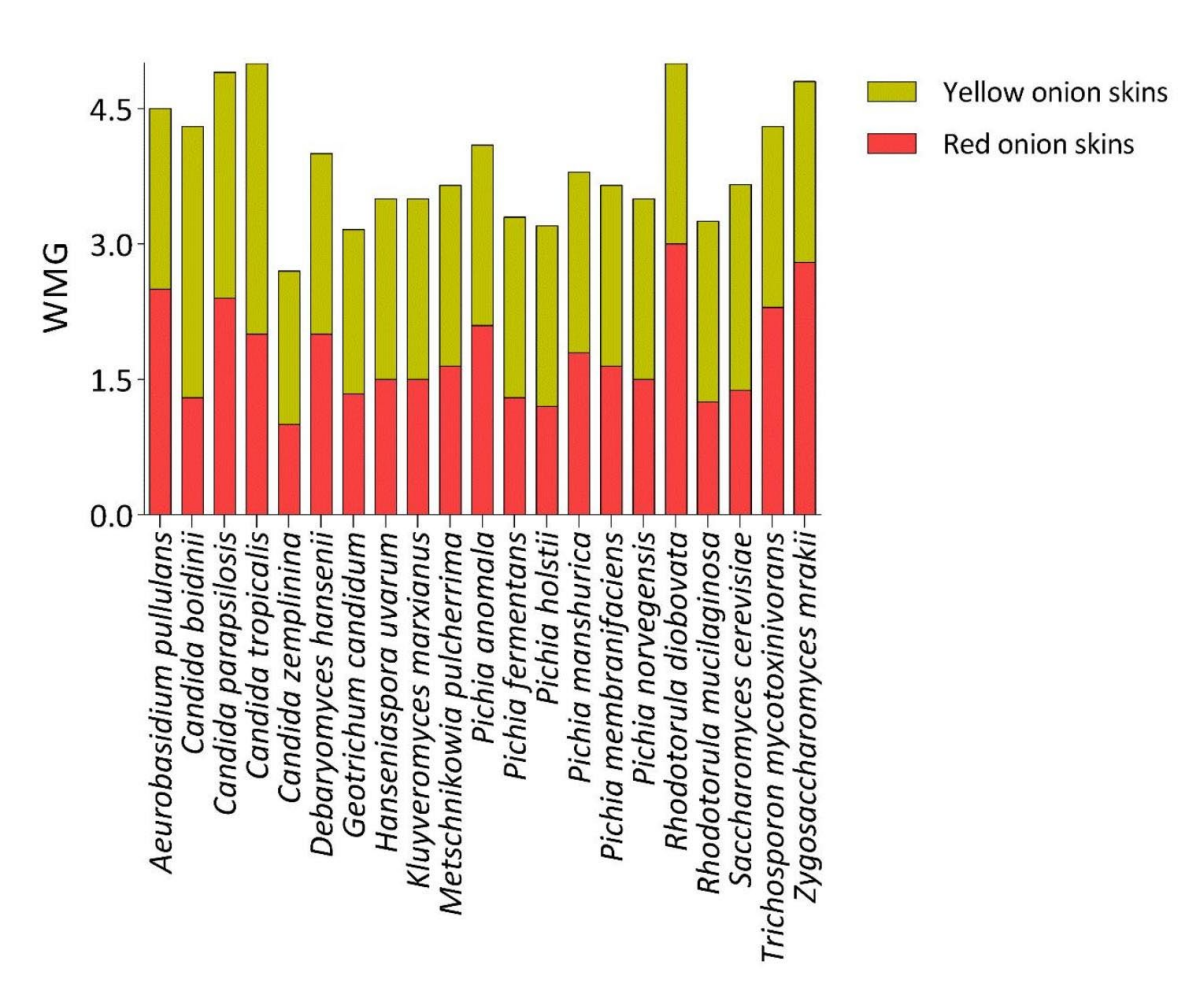
Yeast species ability to grow in presence of different percentages of onion skins homogenate as main nutrient source in plate medium. The reported score is the average value of all the weighted mean growth (WMG) value for the strains belonging to each species

All yeast strains tested were able to grow on the plates containing yellow or red onion skins homogenates, albeit at different levels (Fig. 2 and Table S2). Good results were obtained for strains belonging to species *Aureobasidium pullulans*, *Candida* spp. (*C. boidinii, parapsilosis* and *tropicalis*), *Debaryomyces hansenii*, *Metschnikowia pulcherrima*, *Pichia anomala*, *Rhodotorula diobovata*, *Zygosaccharomyces mrakii*.

The analysis of WMG for yeasts showed values varying between 1 and 2.8 with an average value corresponding to 1.6 for red onion skins. The WMG values varied 1.7 and 3.5 for yellow onion skins, reporting an average value of 2.1. These evidences revealed that yellow onion skins as unique carbon source affected yeast growth lesser than red ones.

At the end of the screening, it was observed that, except for some bacterial strains, yeasts always showed higher WMG values than bacteria.

The bioprospecting approach carried out on 139 total microbial strains growing on semi-defined microbiological media including different percentages of red or yellow skins allowed to select some strains showing the highest WMG values (Table 1) useful for subsequent steps of the procedure.

### Submerged fermentation of onion skins homogenates

Unpasteurized red and yellow onion skins preparations contained initial counts of Enterobacteriaceae (5.7 log_10_ CFU/mL for red, 5 log_10_ CFU/mL for yellow onions), *Bacillus* spp. (4.5 log_10_ CFU/mL for red, 4.3 log_10_ CFU/mL for yellow onions), Staphylococci (3.6 log_10_ CFU/mL), total bacterial count (6 log_10_ CFU/mL), yeasts and moulds (6 log_10_ CFU/mL) for both red and yellow onions.

These results are in accordance with previous observations reporting that onions contamination can occur during handling, processing, and storage by the presence of several microorganism such as *Bacillus cereus* and its spores, lactic acid bacteria, coliforms, *Pseudomonas* sp., yeast, and moulds (i.e. *Aspergillus* sp.) (Pezzutti et al. 2005; Savitha et al. 2022).

Therefore, all fermentation tests were subjected to a heat treatment step to reduce possible interferences on growth and activity of the exogenous starter strains. Indeed, after the heat treatment, no spoilage or potential pathogen microbes were detected in red and yellow onion skins preparations, with the exception of *Bacillus* spp. ranging from 3.6 ± 0.2 and 3.8 ± 0.3 log_10_ CFU/mL in red and yellow onion skins, respectively and Enterobacteriaceae (3.1 ± 0.2 log_10_ CFU/mL) only in red onion skins.

A submerged batch fermentation approach was set up for feedstocks treatment. Few studies have already investigated the possible value of red and yellow onions as the sole substrate for bacterial fermentation (Bisakowski et al. 2007; Kimoto-Nira et al. 2020) and none with yeasts. In this study, the onion skins raw material was homogenized and used to prepare samples to be inoculated with microbial selected strains.

Cultures of the selected bacterial and yeast strains were used to inoculate onion skins homogenates at a final concentration of about 8 log_10_ CFU/mL for bacteria and 7 log_10_ CFU/mL for yeasts to allow the fermentation process starting rapidly and finishing in a limited period of time, by also outcompeting the residual autochthonous microbiota of onion skins, especially spore forming bacteria.

The load of yeasts, Acetic acid bacteria (AAB) and LAB during fermentation was monitored at the 4th and 10th day. As reported in Table 2, viable and culturable counts of AAB and LAB were about 5–6 log_10_ CFU/mL at the 4th day fermentation.

**Table 2:** Microbial changes (log_10_ CFU/ml) occurring in red and yellow onion skins during fermentation (n = 3)

Microbial Species	Medium	Fermentation time (days)		Reference Code	
*Red onions*					
		4	10		
*Acetobacter tropicalis* G25 b	mGYC	6.1 ± 5.4 a	3.15 ± 1.87 b	14	
*Lactiplantibacillus plantarum* TB 11–32	MRS	5.22 ± 4.42 a	3.4 ± 2.62 b	30	
*Metschnikowia pulcherrima* 2047	SDA	6.18 ± 5.36 a	5.36 ± 4.92 a	45	
*Zygosaccharomyces mrakii* CL 30 − 29	SDA	6.48 ± 5.69 a	6.42 ± 5.72 a	57	
***Yellow onions***					
		**4**	**10**		
*Acetobacter tropicalis* G25 b	mGYC	6.31 ± 5.21 a	3.17 ± 2.48 b	14	
*Lactiplantibacillus plantarum* C 180 − 34	MRS	5.15 ± 4.03 a	2.36 ± 1.61 b	28	
*Candida boidinii* A5y	SDA	6.94 ± 5.73 a	6.75 ± 5.25 a	33	
*Saccharomyces cerevisiae* En SC	SDA	6.52 ± 5.28 a	6.05 ± 5.83 a	71	

Values are the means of three independent measurements, ± standard deviation. t-test statistic assay was applied to compare differences between counts of microbial strains at 4 and 10 days of fermentation for each treatment

As already demonstrated during the screening step on plates, the bacterial strains of *Acetobacter tropicalis* and *Lactiplantibacillus plantarum* are expected to live to acid pH present in the onion skins preparations, varying between 3.83 and 4.4 at the initial stage (Table 3). Indeed, the ability of *Acetobacter* sp. to survive in very harsh environment, such as extreme acid conditions in grape must and wines, has been reported (Francois and Kappock 2007; Bartowsky and Henschke 2008). Also, the promising ability to persist in severe conditions and to modify vegetable matrices characterized by high polyphenols content, such as olive mill wastewaters, was previously demonstrated for the selected strain *Acetobacter tropicalis* G25b (Ramires et al. 2020).

**Table 3:** pH and liquid/solid ratio in red and yellow onion skins after 10 days fermentation (n = 3)

Red onion skins				Yellow onion skins			
Strain	Reference code	pH	L/S ratio	Strain	Reference code	pH	L/S ratio
*Acetobacter tropicalis G25b*	14	3.93	3.59 ± 0.51	*Acetobacter tropicalis G25b*	14	5	5,94 ± 0.22
*Lactiplantibacillus plantarum* TB 11–32	30	3.73	3.67 ± 0.44	*Lactiplantibacillus plantarum* C 180 − 34	28	4.5	5,71 ± 0.17
*Metschnikowia pulcherrima* 2047	45	3.91	4.10 ± 0.39	*Candida boidinii* A5y	33	4.96	6,25 ± 0.29^**^
*Zygosaccaromyces mrakii* CL 30 − 29	57	3.97	3.98 ± 0.4	*Saccharomyces cerevisiae* En SC	71	4.7	6,52 ± 0.41^**^
Unfermented^*^	T0	3.83	4.26 ± 0.41	Unfermented^*^	T0	4.4	5,21 ± 0.32

* Measured at the starting point of the test. Values are the means of three independent measurements, ± standard deviation. ANOVA statistic test, followed by Dunn’s multiple comparisons post-test, was performed to compare each fermentation treatment with the control Unfermented (** p < 0.05)

Moreover, the use of *Lactiplantibacillus plantarum* strains has been already proven for a wide range of fermented foods and by-products, remarkably vegetables, where difficult environmental constraints to adapt and survive are present (G-Alegria et al., 2004; Muñoz et al. 2017; Tufariello et al. 2015; Zhou et al. 2020).

At the end of the process the bacterial plate counts reduced (to about 3 log_10_ CFU/mL at 10th day fermentation), this was possible due to the higher concentrations of polyphenols and the limited availability of nutrients in the samples as a result of microbial activities (Table 2). Indeed, the inhibitory effect of different phenolic compounds (such as tannins, terpenes, alkaloids, etc.) partly attributed to their higher concentrations was determined for different LAB (Piekarska-Radzik and Klewicka 2021; García-Ruiz et al. 2012) and *Acetobacter* (Chen et al. 2022) strains.

Concerning yeasts, they maintained high count values during and at the end of the process (about 6–7 log_10_ CFU/mL). These results are not surprising, since they are acidophilic organisms and the more adapted to resist low pH values and the high concentrations of phenols of several challenging niches, such as grape must/wine and olive mill wastes (Bleve et al. 2011, 2016; Liu et al. 2015; Ramires et al. 2020). Also yeasts strains belonging to the species and *Candida boidinii*, *Saccharomyces cerevisiae* and *Metschnikowia pulcherrima* are generally adapted to develop in presence of stress conditions and to produce positive effects on the phenolic compounds (i.e. anthocyanins, hydroxycinnamic acids, flavonols, etc.) (Kelanne et al. 2020; Minnaar et al. 2019; Ramires et al. 2020). In addition, the yeast *Zygosaccharomyces mrakii* strain was isolated and selected from fermented olives and already showed their ability to adapt and survive under difficult environmental constraints (Bleve et al. 2014; Tufariello et al. 2015).

As a preliminary evidence, the liquid/solid ratio showed a statistically significant increase in treated yellow skins (*C. boidinii* A5y code 33 and *S. cerevisiae* code 71, p < 0.05), thus suggesting a potential degrading microbial activity of the plant cell walls of these feedstock (Table 3).

#### Bio-chemical analyses

##### Enzymatic assays

Previous studies demonstrated that bacteria and yeasts produce many different types of enzymes during fermentation, e.g., glycoside hydrolase, cellulose- or xylan-degrading enzymes, and esterase, which break down plant cell walls or starch and depolymerize polysaccharides (Hur et al. 2014).

In comparison with the untreated samples (unfermented T0, Tables 3 and 4), in yellow and red onion skins inoculated with the selected microbial strains, specific enzyme profiles were obtained (Tables 4 and 5). The reported enzyme activities are the sum of the two components within each sample, those deriving from the onion skins tissues and those produced by the microorganisms. A significant increase (p < 0.05) of endo-xylanase activities was detected in all fermented red onion skins samples, whereas the yellow ones showed increased α-amylase activities (p < 0.05). Interestingly, the red and yellow onion skins samples inoculated with the same *Acetobacter tropicalis* G25b strain showed also an increase in lipase and endo-cellulase activities (about 1.4 and 1.6-fold, p < 0.05). The inoculation of *Lactiplantibacillus plantarum* strains was associated to an increased esterase (1.6–2.4 fold, p < 0.05) and lipase (1.3–1.6 fold, p < 0.05) activities in both fermentations, while in presence of *L. plantarum* TB 11–32 and of *L. plantarum* C 180 − 34 strains increased protease and endo-cellulase (1.7 fold, p < 0.05) activities were registered, respectively. Finally, a significant improvement in esterase activities was measured in presence of the yeast *Zygosaccharomyces mrakii* CL 30 − 29 strain in red (1.6 fold, p < 0.05) and of the *Saccharomyces cerevisiae* En SC strain in yellow onion (2.6 fold, p < 0.05) skins.

**Table 4:** Enzyme activities associated with fermented red onion skins

RED ONION SKINS	Esterase	Lipase	Endo-Cellulase	Endo-Xylanase	Amylase	Protease
	mU/mL	U/mL	U/mL	U/mL	U/mL	U/mL
Unfermented (T0)	1.19 ± 0.39 a	34.60 ± 0.76 a	6.71 ± 0.39 a	0.22 ± 0.06 a	15.46 ± 0.56 a	188.54 ± 2.04 a
*Acetobacter tropicalis* *G25 b*	1.09 ± 0.07 a	**58.96 ± 2.40 b**	**9.32 ± 0.56** b	**3.28 ± 0.28 b**	15.77 ± 0.58 a	197.13 ± 1.99 a
*Lactiplantibacillus plantarum* *TB-11-32*	**1.89 ± 0.13 b**	**52.65 ± 1.39 b**	7.03 ± 0.04 a	**1.88 ± 0.21 c**	14.90 ± 0.41 a	**218.64 ± 1.63 b**
*Metschnikowia pulcherrima 2047*	1.37 ± 0.08 a,b	25.63 ± 0.38 a	7.41 ± 0.65 a	**2.26 ± 0.21 c**	13.90 ± 0.40 a	**227.69 ± 1.79 b**
*Zygosaccharomyces mrakii CL 30 − 29*	**1.93 ± 0.10 b**	45.45 ± 2.53 a,b	5.56 ± 0.16 a	**3.06 ± 0.13 b**	16.01 ± 0.28 a	145.57 ± 1.74 a

Data are expressed as mean ± standard deviation (n = 3). Different letters within each column indicate difference in polyphenol concentrations between the samples (p < 0.05) as determined by one-way ANOVA followed by Dunn’s multiple comparisons post-test

**Table 5:** Enzyme activities associated with fermented yellow onion skins

YELLOW ONION SKINS	Esterase	Lipase	Endo- Cellulase	Endo-Xylanase	Amylase	Protease
	mU/mL	U/mL	U/mL	U/mL	U/mL	U/mL
Unfermented (T0)	0.77 ± 0.24 a	113.38 ± 2.78 a	6.63 ± 0.02 a	4.90 ± 0.42 a	6.43 ± 0.12 a	139.10 ± 2.25 a
*Acetobacter tropicalis* *G25 b*	1.10 ± 0.01 a	**157.92 ± 8.24 b**	**10.69 ± 0.75 b**	5.02 ± 0.65 a	**13.76 ± 0.14 b**	135.75 ± 5.09 a
*Lactiplantibacillus plantarum* *C 180 − 34*	**1.85 ± 0.14 b**	**149.56 ± 8.78 b**	**11.56 ± 0.68 b**	1.03 ± 0.50 b	**14.80 ± 0.65 b**	126.08 ± 1.69 a
*Candida boidinii* *A5y*	0.87 ± 0.03 a	123.23 ± 2.78 a,b	**12.09 ± 0.57 b**	1.25 ± 0.23 b	**12.68 ± 0.28 b**	136.53 ± 4.92 a
*Saccharomyces cerevisiae En SC*	**2.01 ± 0.02 b**	68.65 ± 4.42 c	6.63 ± 0.02 a	4.56 ± 0.58 a	**10.97 ± 0.68 b**	87.66 ± 3.49 b

Data are expressed as mean ± standard deviation (n = 3). Data are expressed as mean ± standard deviation (n = 3). Different letters within each column indicate difference in polyphenol concentrations between the samples (p < 0.05) as determined by one-way ANOVA followed by Dunn’s multiple comparisons post-test

Higher enzyme activities in fermented onion skins can be considered as a good indicator of microbial starters active metabolism performed during fermentation of a source different from their original habitat. Indeed, the enzymatic profile expressed in terms of lipase, protease, α-amylase, esterase, endo-cellulase and endo-xylanase activities have been studied in other fermented food products inoculated with selected bacterial and yeast starters (Jolly et al. 2014; Maiorano et al. 2022; Ramires et al. 2022). In addition, these activities were already correlated with the release of bioactive compounds, mainly polyphenols, and with the antioxidant activity, in various plant-based foods (Hur et al. 2014).

#### Polyphenols characterization of fermented onion skins

The qualitative and quantitative characterization of the bioactive compounds mainly polyphenols, identified after microbial pretreatments, was performed by following Folin-Ciocalteu Assay and HPLC-DAD analysis.

After 10 days of fermentation, a significant increase of total phenol content was observed in the red onion skins fermented samples compared to the unfermented control (T0). In particular, the highest content of total polyphenols was present in the sample inoculated with *L. plantarum* TB-11-32 strain (Lp_30), reaching 90.96 ± 3.74 mg GAE/g DW (p < 0.05) (Table 6), although no significant differences were observed among all the fermented samples.

**Table 6:** Total polyphenols content expressed as mg of gallic acid equivalents (GAE) per gram of dry sample (DW) in red and yellow onion skins after 10 days fermentation and unfermented (T0)

Red onion skins			Yellow onion skins		
Microbial strain	**Reference code**	**mg GAE/g DW**	**Microbial strain**	**Reference code**	**mg GAE/g DW**
*Acetobacter tropicalis G25b*	14	86.64 ± 3.38a	*Acetobacter tropicalis G25b*	14	32.30 ± 0.46c
*Lactiplantibacillus plantarum* TB 11–32	30	90.96 ± 3.74a	*Lactiplantibacillus plantarum* C 180 − 34	28	33.08 ± 0.75b
*Metschnikowia pulcherrima* 2047	45	87.54 ± 4.20a	*Candida boidinii* A5y	33	33.85 ± 0.61a
*Zygosaccaromyces mrakii* CL 30 − 29	57	86.30 ± 4.94a	*Saccharomyces cerevisiae* En SC	71	34.24 ± 0.36a
Unfermented	T0	81.11 ± 3.39b	Unfermented	T0	25.46 ± 0.25d

Data are expressed as mean ± standard deviation (n = 3)

Different letters within each column indicate difference in polyphenol concentrations between the samples (p < 0.05) as determined by one-way ANOVA followed by Fisher’s post hoc test

Regarding the yellow onion skins, the fermentation process leaded to an increase of the total polyphenol content in all considered samples and in a range moving from 26.9 to 34.5%, expecially for the sample inoculated with *S. cerevisiae* En SC strain (Sc_71), that showed the highest content (34.24 ± 0.36 mg GAE/g DW, p < 0.05) (Table 6). As was reported for rye bran, brown algae, mulberry, blueberry, yeasts have demonstrated a great potential for the bioactive compounds release; other study demonstrated an increase of total phenolics contents after fermentation by *Saccharomyces* spp. strains (Hur et al. 2014).

Both the red and yellow onion skins extracts have been also characterized for their polyphenol profile by HPLC-DAD analysis.

**Fig. 3:**
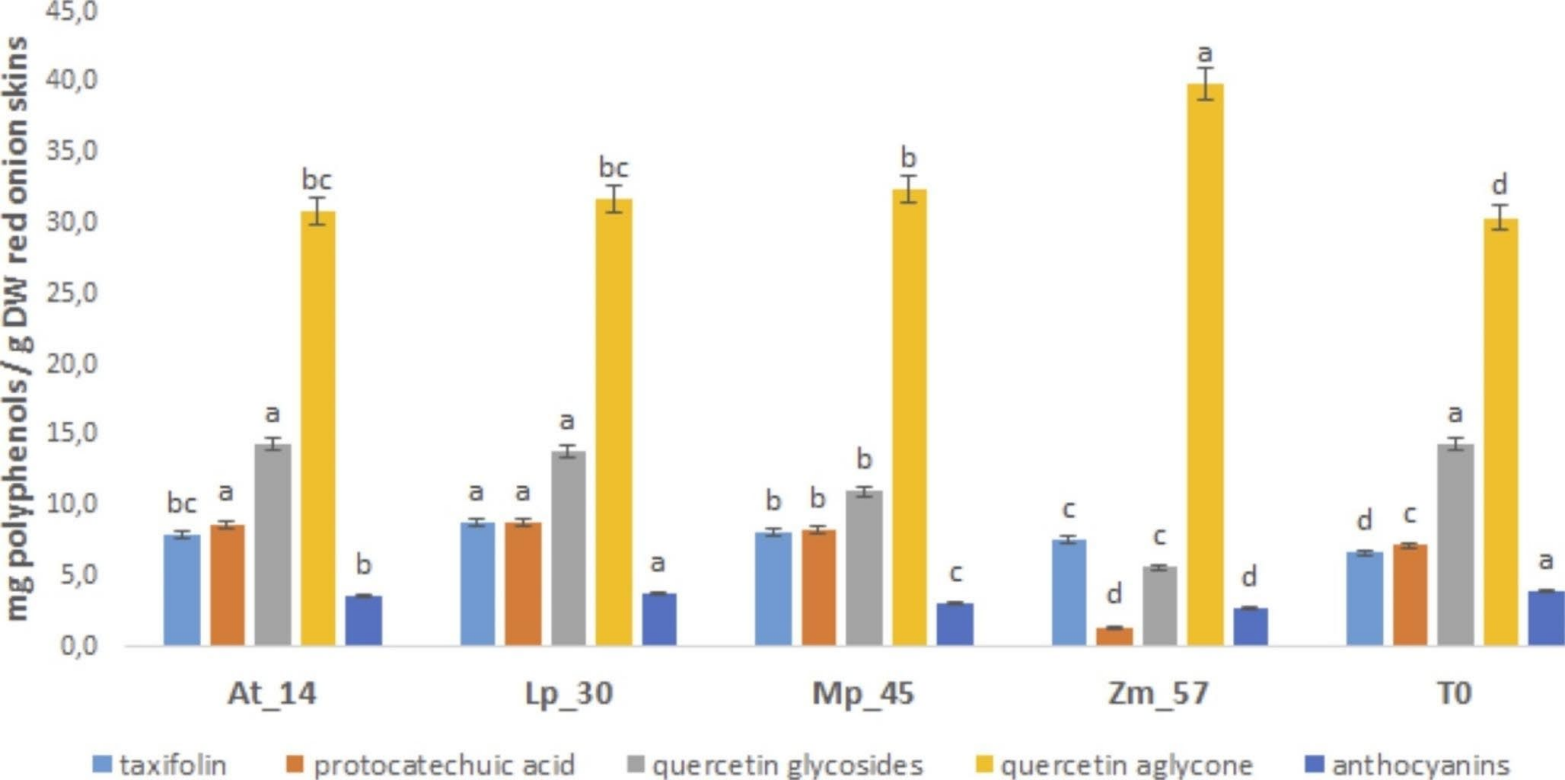
Polyphenols characterization and quantification (mg/g DW) of red onion skins extracts by HPLC-DAD analysis. **14**: *Acetobacter tropicalis* G25b; **30**: *Lactiplantibacillus plantarum* TB 11–32; **45**: *Metschnikowia pulcherrima 2047*; **57**: *Zygosaccharomyces mrakii* CL 30 − 29; **T0**: unfermented onion skins as control. Data are represented as mean ± standard deviation (n = 3). Different letters above the columns indicate significant difference (p < 0.05) among the means of each polyphenol class as determined by one-way ANOVA followed by Fisher’s post hoc test

In the red onion skins, the main polyphenols identified in all the fermented samples belonged to the classes of flavonoids, phenolic acids and anthocyanins (Fig. 3). In particular, the quercetin aglycone was the most abundant, followed by the glycosidic forms of quercetin that for their low relative abundance have been quantified as quercetin glycosides (Fig. 3).

Several research investigation reported the quercetin glycosides conjugated have showed lower antioxidant activity than that quercetin aglycone (Manach et al. 1998). The bioavailability is also affected by the sugary moiety, showing a lower absorption of glycosydes forms respect the aglycone ones (Yang et al. 2012). Hence, the microbial conversion of quercetin glucoside into quercetin aglycone could be a promising strategy to enhance the bioactivity of onion polyphenols. Other identified polyphenols were the dihydroflavonol taxifolin and protocatechuic acid present both for almost 10% of total polyphenols identified, and already identified in onion cultivars (Cattivelli et al. 2023). Recent scientific evidences have reported that the taxifolin rich food can influence brain function also reducing stress (Shinozaki et al. 2023). The anthocyanins (cyanidine derivatives) were about 5% of the total polyphenols identified and they did not undergo variations probably due to the acidic environment of fermented products. Notewhortly to underline the significant differences among the identified polyphenols in the fermented samples in comparison with the unfermented control (T0). In particular, in the samples 57 (fermented by yeast *Z. mrakii* CL 30 − 29) the quercetin aglycone increase of about 25% (significant difference p < 0.05) with a simultaneous reduction of glycosides quercetins, probably due to the selective deglycosylation reaction occurred on the glycosylic moiety of quercetin glycoside, as reported for *S. cerevisiae* in onion, by Chung et al. (2011) (Fig. 3).

**Fig. 4:**
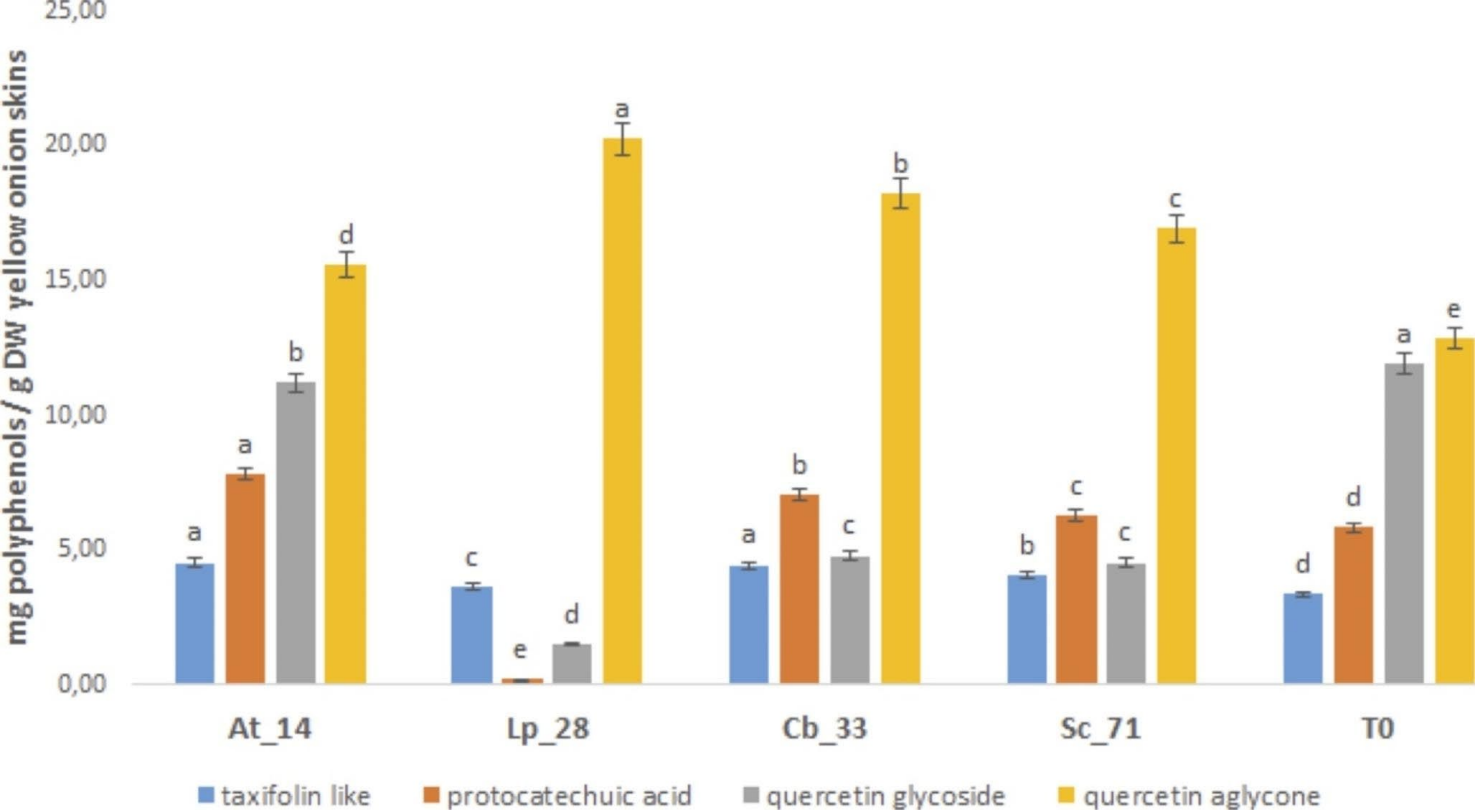
Polyphenolic characterization and quantification (mg/g DW) in yellow onion skins extracts by HPLC-DAD analysis. **14**: *Acetobacter tropicalis* G25b; **28**: *Lactiplantibacillus plantarum* C 180 − 34; **33**: *Candida boidinii* A5y; **71**: *Saccharomyces cerevisiae* En SC; **T0**: unfermented onion skins as control. Data are represented as mean ± standard deviation (n = 3). Different letters above the columns indicate significant difference (p < 0.05) among the means of each polyphenol class as determined by one-way ANOVA followed by Fisher’s post hoc test

Taxifolin, protocatechuic acid and glycosylated and aglycone quercetins have been identified also in fermented yellow onion skins by HPLC-DAD analysis (Fig. 4). The results showed that the microbial treatment modify significantly the relative abundance of the identified polyphenols (significant difference p < 0.05) compared to the unfermented sample (T0).

In particular, the fermented sample with *A. tropicalis* G25b (code 14) showed the higher amount of taxifolin and protocatechuic acid (4.54 and 7.82 mg/gDW, respectively) (p < 0.05); instead, the sample fermented with *L. plantarum* C 180 − 34 (code 28), underlined the greater increase of quercetin aglycone of about 60% (p < 0.05) compared to the unfermented control.

The reported effect, already highlighted in the red onion skins, can be attributed to lactic acid bacteria (LAB) that can survive to the high polyphenols amount also modificating, by action of a wide enzymatic activities and of the pH, the complex phenolics structure increasing the amount of single compound, also enhancing the health promoting effects of fermented food (Khubber et al. 2022; Kimoto-Nira et al. 2020; Tsangalis et al. 2002). In addition, in the same sample, the protocatechuic acid underwent to a strong degradation probably for the specific microbial metabolism of the selected strain *L. plantarum* C 180 − 34 (Othman et al. 2009).

### Principal component analysis

**Fig. 5:**
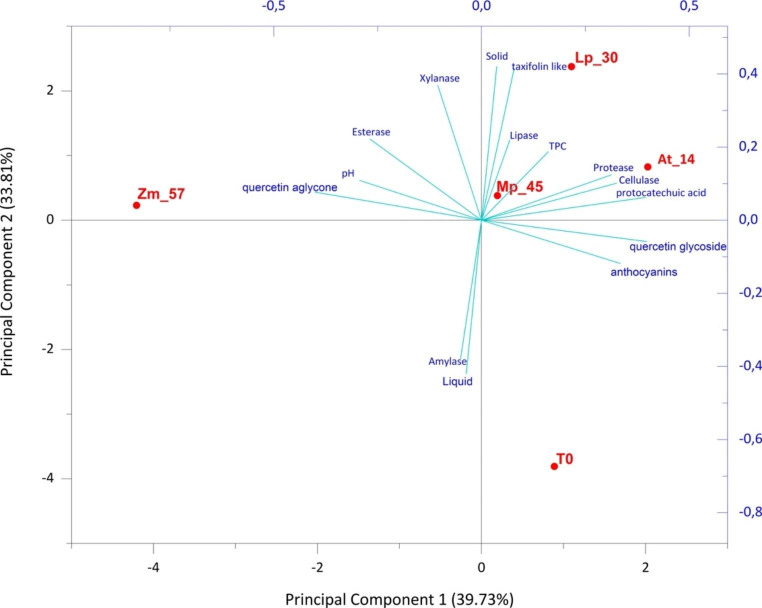
Score scatter plot of PCA model performed on parameters associated with all red onion skin fermented samples. PCA variables were the data obtained from the analysis of values of chemical composition, nutritional traits, enzyme-associated activities. **At_14**: *Acetobacter tropicalis* G25b; **Lp_30**: *Lactiplantibacillus plantarum* TB 11–32; **Mp_45**: *Metschnikowia pulcherrima 2047*; **Zm_57**: *Zygosaccharomyces mrakii* CL 30 − 29; **T0**: unfermented onion skins as control

Principal component analysis was used to apply an unsupervised approach for analyzing the existing relationship among the chemical, physical and biochemical datasets produced during the study and the tested samples of unfermented and fermented red and yellow onion skins. A total variance of 73.54% was observed for red onion skins (Fig. 5).

The two planes made using the first two principal components (PCs) showed clustering of the red onion skins samples into three groups. One group, consisting of samples treated with the bacterial strain *L. plantarum* TB 11–32 (Lp_30), with the *A. tropicalis* G25b (At_14), and with the yeast strain *M. pulcherrima* 2047 (Mp_45), were closely associated to the parameters total phenolic content (TPC), taxifolin and protocatechuic acid, and, lipase, cellulase and protease activities.

The second group consisted of the sample treated with *Z. mrakii* CL 30 − 29 (Zm_57) located in the opposite portion of the plane (and mainly associated to pH, quercetin aglycone, esterase and xylanase activities). The untreated sample (T0) was located on the negative semi-axis of the first component, discriminating it from all the other treatments.

**Fig. 6:**
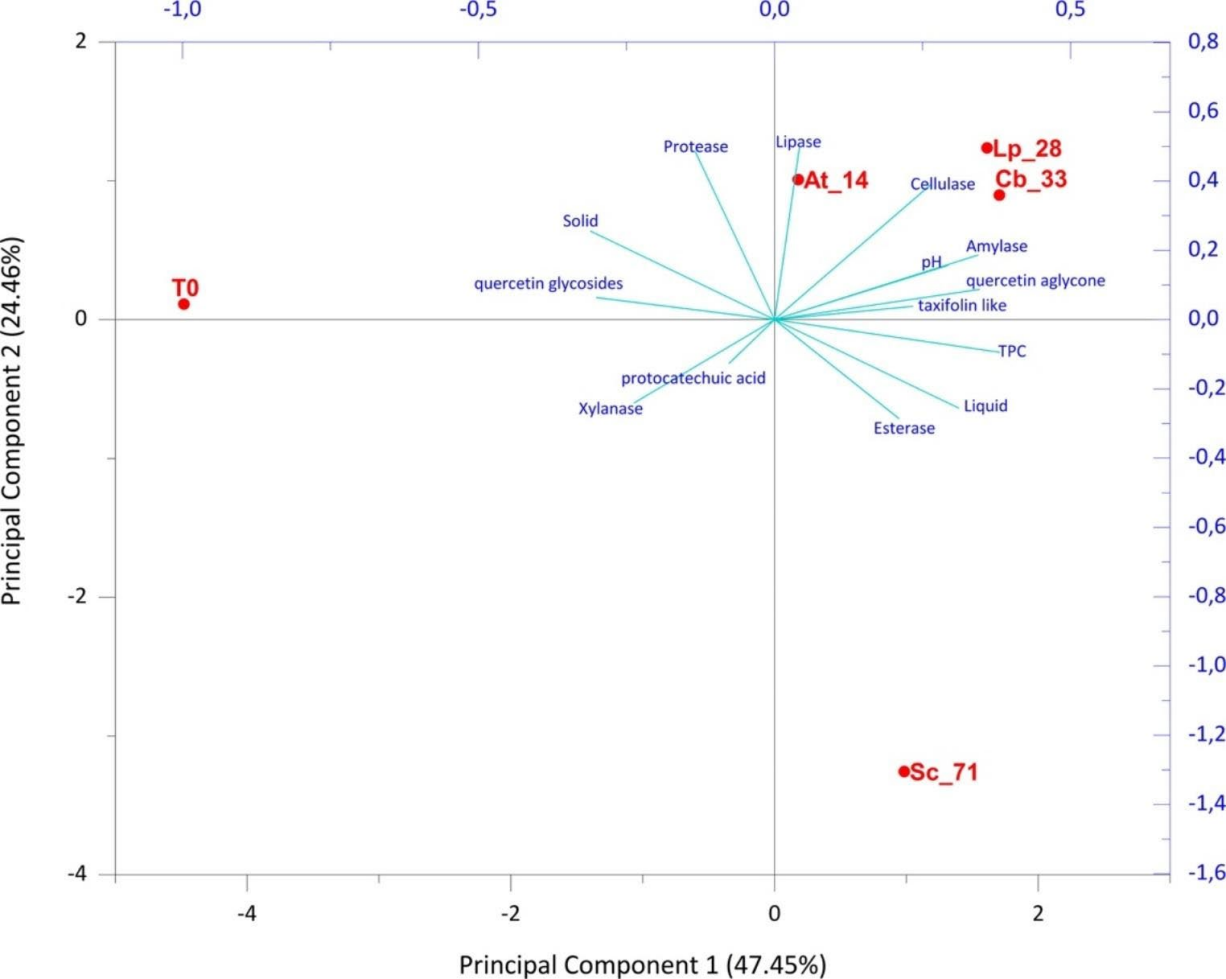
Score scatter plot of PCA model performed on parameters associated with all yellow onion skin fermented samples. PCA variables were the data obtained from the analysis of values of chemical composition, nutritional traits, enzyme-associated activities. **At_14**: *Acetobacter tropicalis* G25b; **Lp_28**: *Lactiplantibacillus plantarum* C 180 − 34; **Cb_33**: *Candida boidinii* A5y; **Sc_71**: *Saccharomyces cerevisiae* En SC; **T0**: unfermented onion skins as control

Concerning yellow onion skins, a total variance of 71.91% was reported (Fig. 6) and also in this case, three groups were identified.

The samples treated with the inoculated bacterial and yeast strains were clearly separated by the untreated one (T0). The samples treated with yeast strain *C. boidinii* A5y (Cb_33) and the bacterial strains *L. plantarum* C 180 − 34 (Lp_28) and *A. tropicalis* G25b (At_14) grouped together and were characterized by quercetin aglycone, taxifolin, pH, lipase, cellulase and a-amylase activities. The second group represented by *S. cerevisiae* En SC (Sc_71) treated sample was mainly associated to TPC and esterase activity.

The choice of microorganism to be used in the fermentation process depends on the desired end products and/or final applications of the treated samples. However, this study suggests that mostly the release yields of bioactive compounds (i.e. polyphenols) and the production of microbial enzymes of biotechnological interest, can be improved with a suitable choice of microorganism to re-use the byproduct onion skins. Additionally, the preliminary evidences can open the opportunity to further study the possible correlation of microbial hydrolytic enzyme activities (such as α-amylase, cellulase, etc.) to the TPC and then in the release of phenolic, as already demonstrated by Chen et al. (2020) for oat.

## Conclusions

In the present work, a new procedure for screening microbial biodiversity able to improve the recovery of bioactive compounds and the production of enzymes of biotechnological interest from the lignocellulosic material onion skins was developed. The fermentation was performed using different bacterial (*Lactiplantibacillus* and *Acetobacter* spp.) and yeast (*Saccharomyces, Metschnikowia* and *Candida* spp.) strains. The resulting products exhibited different levels of polyphenols and enzymatic activities. In fermented yellow onion skins, the release of total phenolic compounds was remarkable, as it was increased of about 26.9–34.5% in comparison with the untreated sample. The treatments of yellow onion skins with *L. plantarum* C 180 − 34 and *S. cerevisae* En SC were the most promising processes in terms of recovery of quercetin aglycone as well as of associated interesting enzymes activities. A weaker effect, but still significant, was observed for red onion skin sample treated with *L. plantarum* TB 11–32 (> 11% TPC than untreated sample). However, the treatment with the yeast strain *Zygosaccharomyces mrakii* CL 30 − 29 enhanced the quercetin aglycone content of about 25% of the initial raw material.

By this way, the microbial pre-treatment driven by selected bacterial and yeast strains here developed can be considered as a new method, alternative to the broadly recognized traditional physical and chemical approaches, for enhancing the extraction of polyphenols from onion skins feedstock by a sustainable and low-cost route. The peculiar microbial metabolic activities were also able to significantly enrich the recovery of quercetin aglycone, which is a compound greatly requested for its high antioxidant activity. The here set-up bio-process combination with other technologies and scale-up optimization could further enhance bioactive compounds yield and bioactivity.

## Electronic supplementary material

Below is the link to the electronic supplementary material.

Supplementary Material 1

## Data Availability

All data generated or analysed during this study are included in this published article and its supplementary information file.

## References

[CR1] Barcelos MC, Ramos CL, Kuddus M, Rodriguez-Couto S, Srivastava N, Ramteke PW, Mishra PK, Molina G (2020). Enzymatic potential for the valorization of agro-industrial by-products. Biotechnol Lett.

[CR2] Bartowsky EJ, Henschke PA (2008). Acetic acid bacteria spoilage of bottled red wine—a review. Int J Food Microbiol.

[CR3] Bei Q, Chen G, Lu F, Wu S, Wu Z (2018). Enzymatic action mechanism of phenolic mobilization in oats (Avena sativa L.) during solid-state fermentation with *Monascus anka*. Food Chem.

[CR4] Benítez V, Mollá E, Martín-Cabrejas MA, Aguilera Y, López-Andréu FJ, Cools K, Terry LA, Esteban RM (2011). Characterization of industrial onion wastes (*Allium cepa* L.): dietary fibre and bioactive compounds. Plant Foods Hum Nutr.

[CR5] Benito-Román Ó, Blanco B, Sanz MT, Beltrán S (2020). Subcritical water extraction of phenolic compounds from onion skin wastes (*Allium cepa cv. Horcal*): effect of temperature and solvent properties. Antioxidants.

[CR6] Bisakowski B, Atwal AS, Gardner N, Champagne CP (2007). Effect of lactic acid fermentation of onions (*Allium cepa*) on the composition of flavonol glucosides. Int J Food Sci.

[CR9] Bleve G, Lezzi C, Chiriatti MA, D’Ostuni I, Tristezza M, Di Venere D, Sergio L, Mita G, Grieco F (2011). Selection of non-conventional yeasts and their use in immobilized form for the bioremediation of olive oil mill wastewaters. Bioresour Technol.

[CR8] Bleve G, Tufariello M, Durante M, Perbellini E, Ramires FA, Grieco F, Cappello MS, De Domenico S, Mita G, Tasioula-Margari M, Logrieco AF (2014). Physico-chemical and microbiological characterization of spontaneous fermentation of Cellina di Nardò and Leccino table olives. Front Microbiol.

[CR7] Bleve G, Tufariello M, Vetrano C, Mita G, Grieco F (2016). Simultaneous alcoholic and malolactic fermentations by *Saccharomyces cerevisiae* and *Oenococcus oeni* cells co-immobilized in alginate beads. Front Microbiol.

[CR10] Carmona-Cabello M, Garcia IL, Leiva-Candia D, Dorado MP (2018). Valorization of food waste based on its composition through the concept of biorefinery. Curr Opin Green Sustain Chem.

[CR11] Cattivelli A, Nissen L, Casciano F, Tagliazucchi D, Gianotti A (2023). Impact of cooking methods of red-skinned onion on colon metabolic transformation of phenolic compounds and gut microbiota changes. Food Funct.

[CR12] Celano R, Docimo T, Piccinelli AL, Gazzerro P, Tucci M, Di Sanzo R, Carabetta S, Campone L, Russo M, Rastrelli L (2021). Onion Peel: turning a Food Waste into a resource. Antioxidants.

[CR15] Chen G, Liu Y, Zeng J, Tian X, Bei Q, Wu Z (2020). Enhancing three phenolic fractions of oats (*Avena sativa L*.) and their antioxidant activities by solid-state fermentation with *Monascus anka* and *Bacillus subtilis*. J Cereal Sci.

[CR14] Chen C, Wu S, Li Y, Huang Y, Yang X (2022). Effects of different acetic acid bacteria strains on the bioactive compounds, volatile compounds and antioxidant activity of black tea vinegar. LWT.

[CR13] Cheng A, Chen X, Jin Q, Wang W, Shi J, Liu Y (2013). Comparison of phenolic content and antioxidant capacity of red and yellow onions. Czech J Food Sci.

[CR16] Chung DM, Chung YC, Maeng PJ, Chun HK (2011). Regioselective deglycosylation of onion quercetin glucosides by *Saccharomyces cerevisiae*. Biotechnol Lett.

[CR17] Cicco N, Lanorte MT, Paraggio M, Viggiano M, Lattanzio V (2009). A reproducible, rapid and inexpensive folin–ciocalteu micro-method in determining phenolics of plant methanol extracts. Microchem J.

[CR18] D’Antuono I, Bruno A, Linsalata V, Minervini F, Garbetta A, Tufariello M, Mita G, Logrieco AF, Bleve G, Cardinali A (2018). Fermented apulian table olives: Effect of selected microbial starters on polyphenols composition, antioxidant activities and bioaccessibility. Food Chem.

[CR19] Dey TB, Chakraborty S, Jain KK, Sharma A, Kuhad RC (2016). Antioxidant phenolics and their microbial production by submerged and solid state fermentation process: a review. Trends Food Sci Technol.

[CR20] FAOSTAT. FAO Statistics Division (2018) Available online: http//www.fao.org/faostat/en/#data (accessed on 17 April 2023)

[CR21] Francois JA, Kappock TJ (2007). Alanine racemase from the acidophile *Acetobacter aceti*. Protein Expr Purif.

[CR22] G-Alegría E, López I, Ruiz JI, Sáenz J, Fernández E, Zarazaga M, Dizy M, Torres C, Ruiz-Larrea F (2004). High tolerance of wild *Lactobacillus plantarum* and *Oenococcus oeni* strains to lyophilisation and stress environmental conditions of acid pH and ethanol. FEMS Microbiol Lett.

[CR23] Ganguly P, Khan A, Das P, Bhowal A (2021). Cellulose from lignocellulose kitchen waste and its application for energy and environment: bioethanol production and dye removal. Indian Chem Eng.

[CR24] García-Ruiz A, Cueva C, González-Rompinelli EM, Yuste M, Torres M, Martín-Álvarez PJ, Bartolomé B, Moreno-Arribas MV (2012). Antimicrobial phenolic extracts able to inhibit lactic acid bacteria growth and wine malolactic fermentation. Food Control.

[CR25] Gontard N, Sonesson U, Birkved M, Majone M, Bolzonella D, Celli A, Angellier-Coussy H, Jang GW, Verniquet A, Broeze J, Schaer B, Batista AP, Sebok A (2018). A research challenge vision regarding management of agricultural waste in a circular bio-based economy. Crit Rev Environ Sci Technol.

[CR27] Gulsunoglu-Konuskan Z, Kilic-Akyilmaz M (2022). Microbial Bioconversion of Phenolic Compounds in agro-industrial wastes: a review of mechanisms and effective factors. J Agric Food Chem.

[CR26] Gulsunoglu-Konuskan Z, Karbancioglu-Guler F, Kilic-Akyilmaz M (2021). Development of a bioprocess for production of ellagic acid from chestnut (*Castanea sativa* Mill.) Waste by fermentation with *aspergillus spp*. Food Biosci.

[CR28] Hommel RK (2014) *Acetobacter*, in Batt C.A, Tortorello ML. (Academic Press) Encyclopedia of Food Microbiology (2th ed. pp. 3–10) ISBN 9780123847331 10.1016/B978-0-12-384730-0.00001-X

[CR29] Hur SJ, Lee SY, Kim YC, Choi I, Kim GB (2014). Effect of fermentation on the antioxidant activity in plant-based foods. Food Chem.

[CR30] Jolly NP, Varela C, Pretorius IS (2014). Not your ordinary yeast: non-saccharomyces yeasts in wine production uncovered. FEMS Yeast Res.

[CR31] Kelanne N, Yang B, Liljenbäck L, Laaksonen O (2020). Phenolic compound profiles in alcoholic black currant beverages produced by fermentation with *Saccharomyces* and non-*Saccharomyces* yeasts. J Agric Food Chem.

[CR34] Khubber S, Marti-Quijal FJ, Tomasevic I, Remize F, Barba FJ (2022). Lactic acid fermentation as a useful strategy to recover antimicrobial and antioxidant compounds from food and by-products. Curr Opin Food.

[CR32] Kim HM, Choi IS, Lee S, Yang JE, Jeong SG, Park JH, Hwang IM, Chun HH, Wi SG, Kim JC, Park HW (2019). Biorefining process of carbohydrate feedstock (agricultural onion waste) to acetic acid. ACS omega.

[CR33] Kimoto-Nira H, Ohashi Y, Amamiya M, Moriya N, Ohmori H, Sekiyama Y (2020). Fermentation of onion (*Allium cepa* L.) peel by lactic acid bacteria for production of functional food. J Food Meas Charact.

[CR36] Liu X, Jia B, Sun X, Ai J, Wang L, Wang C, Zhao F, Zhan J, Huang W (2015). Effect of initial pH on growth characteristics and fermentation properties of *Saccharomyces cerevisiae*. J Food Sci.

[CR35] Lopes DB, Fraga LP, Fleuri LF, Macedo GA (2011). Lipase and esterase: to what extent can this classification be applied accurately?. Food Sci Technol.

[CR37] Maiorano G, Ramires FA, Durante M, Palamà IE, Blando F, De Rinaldis G, Perbellini E, Patruno V, Gadaleta Caldarola C, Vitucci S, Mita G, Bleve G (2022). The controlled Semi-Solid Fermentation of Seaweeds as a strategy for their stabilization and new food applications. Foods.

[CR38] Manach C, Morand C, Crespy V, Demigné C, Texier O, Régérat F, Rémésy C (1998). Quercetin is recovered in human plasma as conjugated derivatives which retain antioxidant properties. FEBS Lett.

[CR39] Minnaar PP, Du Plessis HW, Jolly NP, Van Der Rijst M, Du Toit M (2019) Non-*Saccharomyces* yeast and lactic acid bacteria in co-inoculated fermentations with two Saccharomyces cerevisiae yeast strains: a strategy to improve the phenolic content of Syrah wine. Food Chem X 4100070. 10.1016/j.fochx.2019.100070 10.1016/j.fochx.2019.100070 PMC6806450 31656955

[CR40] Moyano FJ, Diaz M, Alarcón FJ, Sarasquete MC (1996). Characterization of digestive enzyme activity during larval development of gilthead seabream (*Sparus aurata*). Fish Physiol Biochem.

[CR41] Munir MT, Kheirkhah H, Baroutian S, Quek SY, Young BR (2018). Subcritical water extraction of bioactive compounds from waste onion skin. J Clean Prod.

[CR42] Muñoz R, de las Rivas B, López de Felipe F, Reverón I, Santamaría L, Esteban-Torres M, Curiel JA, Rodríguez H, Landete JM (2017) Biotransformation of Phenolics by Lactobacillus plantarum in Fermented Foods, In Frias, J., Martinez-Villaluenga, C., Peñas E. (Academic Press) Fermented Foods in Health and Disease Prevention (pp. 63–83) ISBN 9780128023099, 10.1016/B978-0-12-802309-9.00004-2

[CR43] Niu B, Paulson JN, Zheng X, Kolter R (2017). Simplified and representative bacterial community of maize roots. Proc Natl Acad Sci USA.

[CR44] Oren A, Garrity GM (2021). Valid publication of the names of forty-two phyla of prokaryotes. Int J Syst Evol Microbiol.

[CR45] Othman NB, Roblain D, Chammen N, Thonart P, Hamdi M (2009). Antioxidant phenolic compounds loss during the fermentation of Chétoui olives. Food Chem.

[CR46] Park SY, Kim JT, Kang SG, Woo JH, Lee JH, Choi HT, Kim SJ (2007). A new esterase showing similarity to putative dienelactone hydrolase from a strict marine bacterium, Vibrio sp. GMD509. Appl Microbiol Biotechnol.

[CR47] Pezzutti A, Marucci PL, Sica MG, Matzkin MR, Croci CA (2005). Gamma-ray sanitization of argentinean dehydrated garlic (*Allium sativum* L.) and onion (*Allium cepa* L.) products. Food Res Int.

[CR48] Piekarska-Radzik L, Klewicka E (2021). Mutual influence of polyphenols and *Lactobacillus* spp. bacteria in food: a review. Eur Food Res Technol.

[CR49] Poe NE, Yu D, Jin Q, Ponder MA, Stewart AC, Ogejo JA, Wang H, Huang H (2020). Compositional variability of food wastes and its effects on acetone-butanol-ethanol fermentation. Waste Manage.

[CR51] Ramires FA, Durante M, Maiorano G, Migoni D, Rampino P, Fanizzi FP, Perrotta C, Mita G, Grieco F, Bleve G (2020). Industrial scale bio-detoxification of raw olive mill wastewaters by the use of selected microbial yeast and bacterial strains to obtain a new source for fertigation. J Environ Manage.

[CR50] Ramires FA, Bleve G, De Domenico S, Leone A (2022). Combination of solid state and submerged fermentation strategies to produce a New Jellyfish-Based food. Foods.

[CR52] Ren F, Zhou S (2021). Phenolic Components and Health Beneficial Properties of onions. Agriculture.

[CR53] Roldán E, Sánchez-Moreno C, De Ancos B, Cano MP (2008). Characterisation of onion (*Allium cepa* L.) by-products as food ingredients with antioxidant and antibrowning properties. Food Chem.

[CR54] Sagar NA, Pareek S (2020). Dough rheology, antioxidants, textural, physicochemical characteristics, and sensory quality of pizza base enriched with onion (*Allium cepa* L.) skin powder. Sci Rep.

[CR55] Sagar NA, Pareek S, Gonzalez-Aguilar GA (2020). Quantification of flavonoids, total phenols and antioxidant properties of onion skin: a comparative study of fifteen indian cultivars. J Food Sci Technol.

[CR56] Santiago B, Calvo AA, Gullon B, Feijoo G, Moreira MT, Gonzalez-Garcia S (2020). Production of flavonol quercetin and fructooligosaccharides from onion (*Allium cepa* L.) waste: an environmental life cycle approach. J Chem Eng.

[CR57] Savitha S, Chakraborty S, Thorat BN (2022). Microbial Contamination and Decontamination of Onion and its products. Appl Food Res.

[CR58] Sáyago-Ayerdi SG, Venema K, Tabernero M, Sarriá B, Bravo L, Mateos R (2021). Bioconversion of polyphenols and organic acids by gut microbiota of predigested Hibiscus sabdariffa L. calyces and Agave (A. tequilana Weber) fructans assessed in a dynamic in vitro model (TIM-2) of the human colon. Food Res Int.

[CR59] Sharma K, Mahato N, Nile SH, Lee ET, Lee YR (2016). Economical and environmentally-friendly approaches for usage of onion (*Allium cepa* L.) waste. Food Funct.

[CR60] Shinozaki F, Kamei A, Shimada K, Matsuura H, Shibata T, Ikeuchi M, Yasuda K, Oroguchi T, Kishimoto N, Takashimizu S, Nishizaki Y, Abe K (2023). Ingestion of taxifolin-rich foods affects brain activity, mental fatigue, and the whole blood transcriptome in healthy young adults: a randomized, double-blind, placebo-controlled, crossover study. Food Funct.

[CR61] Slama N, Mankai H, Limam F (2021). *Streptomyces tunisiensis* DSM 42037 mediated bioconversion of ferulic acid released from barley bran. World J Microbiol Biotechnol.

[CR62] Tsangalis D, Ashton JF, McGill AEJ, Shah NP (2002). Enzymic transformation of isoflavone phytoestrogens in soymilk by β-glucosidase‐producing bifidobacteria. J Food Sci.

[CR63] Tufariello M, Durante M, Ramires FA, Grieco F, Tommasi L, Perbellini E, Falco V, Tasioula-Margari M, Logrieco AF, Mita G, Bleve G (2015). New process for production of fermented black table olives using selected autochthonous microbial resources. Front Microbiol.

[CR64] Walter HE. Proteinases: Methods with hemoglobin, casein and azocoll as substrates in: Methods of Enzymatic Analysis, Vol. 5, Bergmeyer, Bermeyer HU (1984) J

[CR65] Yang EJ, Kim SI, Park SY, Bang HY, Jeong JH, So JH, Rhee IK, Song KS (2012). Fermentation enhances the in vitro antioxidative effect of onion (*Allium cepa*) via an increase in quercetin content. Food Chem Toxicol.

[CR66] Yin L, Zhang Y, Wu H, Wang Z, Dai Y, Zhou J, Liu X, Dong M, Xia X (2020). Improvement of the phenolic content, antioxidant activity, and nutritional quality of tofu fermented with *Actinomucor elegans*. LWT.

[CR67] Zhou Y, Wang R, Zhang Y, Yang Y, Sun X, Zhang Q, Yang N (2020). Biotransformation of phenolics and metabolites and the change in antioxidant activity in kiwifruit induced by *Lactobacillus plantarum* fermentation. J Sci Food Agric.

