# Supplementary material for: Liquid submerged fermentation by selected microbial strains for onion skins valorization and its effects on polyphenols
Source: World J Microbiol Biotechnol. 2023 Jul 26;39(10):258. doi: 10.1007/s11274-023-03708-y (PMC10371881; doi:10.1007/s11274-023-03708-y)
Supplement: Supplementary file 1 — Supplementary Material 1 [file 11274_2023_3708_MOESM1_ESM.docx]

**Table S1.** Bacterial Strain screened against red and yellow onion skins-based media. The Reference code is an internal code used to univocally identify the strain used in this study.

| Genus or Species | Strain | Reference code | Red onion skins | | | | Yellow onion skins | | | |
| --- | --- | --- | --- | --- | --- | --- | --- | --- | --- | --- |
|  |  |  | **15%** | **20%** | **25%** | **WMG** | **15%** | **20%** | **25%** | **WMG** |
| *Acetobacter tropicalis* | G25 b | 14 | 3 | 4 | 4 | 3,8 | 3 | 3 | 4 | 3,5 |
| *Agrobacterium tumefaciens* | LR1-3 | LR1-3 | 1 | 0 | 1 | 0,7 | 0 | 0 | 0 | 0 |
| *Bacillus amyloliquefaciens* | DSM7 | 2 | 1 | 1 | 1 | 1 | 1 | 0 | 0 | 0,2 |
| *Bacillus amyloquefaciens* | MS3 | 19 | 1 | 1 | 0 | 0,5 | 0 | 0 | 0 | 0 |
| *Bacillus megaterium* |  | 20 | 1 | 1 | 1 | 1 | 0 | 0 | 0 | 0 |
| *Bacillus licheniformis* | DSM 13 | 4 | 1 | 1 | 0 | 0,5 | 0 | 0 | 0 | 0 |
| *Bacillus pumilus* | DSM 27 | 5 | 1 | 1 | 0 | 0,5 | 0 | 0 | 1 | 0,5 |
| *Bacillus simplex* | F2b | 11 | 0 | 1 | 0 | 0,3 | 1 | 1 | 1 | 1 |
| *Bacillus simplex* | R21 b | 15 | 1 | 2 | 0 | 0,8 | 1 | 1 | 1 | 1 |
| *Bacillus simplex* | R23 b | 16 | 3 | 2 | 0 | 1,2 | 1 | 1 | 1 | 1 |
| *Bacillus spizizenii* | ATCC663 | 1 | 1 | 1 | 1 | 1 | 0 | 0 | 0 | 0 |
| *Bacillus subtilis* | DSM 10 | 3 | 1 | 1 | 0 | 0,5 | 0 | 0 | 0 | 0 |
| *Bacillus spp* | DSM 2034 | 6 | 1 | 1 | 0 | 0,5 | 1 | 1 | 1 | 1 |
| *Bacillus spp.* | RAD F1 | RAD F1 | 1 | 2 | 1 | 1,3 | 0 | 0 | 0 | 0 |
| *Bacillus spp.* | RAD F 2 | RAD F2 | 1 | 2 | 0 | 0,8 | 0 | 0 | 0 | 0 |
| *Bacillus spp.* | MT5 | MT5 | 0 | 0 | 0 | 0 | 0 | 0 | 0 | 0 |
| *Dickeya dadantis* | LMG 25991 | LMG 25991 | 0 | 0 | 0 | 0 | 0 | 0 | 0 | 0 |
| *Enterbacter hormaechei* | MR1-1 | MR1-1 | 3 | 3 | 3 | 3 | 0 | 0 | 0 | 0 |
| *Enterobacter asburiae* | MR1-2 | MR1-2 | 1 | 0 | 0 | 0,2 | 0 | 0 | 0 | 0 |
| *Enterobacter asburiae* | RP2 | RP2 | 3 | 3 | 2 | 2,5 | 0 | 0 | 0 | 0 |
| *Enterobacter asburiae* | LP1 | LP1 | 1 | 0 | 0 | 0,2 | 0 | 0 | 0 | 0 |
| *Enterobacter asburiae* | LR2-1 | LR2-1 | 3 | 3 | 3 | 3 | 0 | 0 | 0 | 0 |
| *Erwinia persicina* | A46EP | A46EP | 0 | 0 | 0 | 0 | 0 | 0 | 0 | 0 |
| *Erwinia toletana* | MP3 | MP3 | 1 | 0 | 0 | 0,2 | 0 | 0 | 0 | 0 |
| *Hafnia alvei* | C1 | C1 | 1 | 0 | 0 | 0,2 | 0 | 0 | 0 | 0 |
| *Lactiplantibacillus plantarum* | C180-11 | 27 | 1 | 1 | 0 | 0,5 | 2 | 2 | 2 | 2 |
| *Lactiplantibacillus plantarum* | C 180-34 | 28 | 1 | 2 | 1 | 1,3 | 2 | 2 | 2 | 2 |
| *Lactiplantibacillus plantarum* | TB-11-32 | 30 | 3 | 2 | 2 | 2,3 | 2 | 2 | 2 | 2 |
| *Lactiplantibacillus plantarum* | BC-T3-35 | 32 | 1 | 2 | 1 | 1,3 | 2 | 2 | 2 | 2 |
| *Leuconostoc mesenteroides* | KT 5-1 | 26 | 0 | 1 | 1 | 0,8 | 2 | 2 | 2 | 2 |
| *Levilactobacillus brevis* | TM-2_10_lac | 29 | 1 | 1 | 0 | 0,5 | 2 | 2 | 2 | 2 |
| *Levilactobacillus brevis* | LMG 11437 | 31 | 1 | 1 | 1 | 1 | 2 | 2 | 2 | 2 |
| *Paenibacillus agaridevorans* | C12b | 10 | 0 | 1 | 0 | 0,3 | 1 | 1 | 1 | 1 |
| *Paenibacillus albidus* | E30b | 12 | 0 | 1 | 0 | 0,3 | 1 | 1 | 1 | 1 |
| *Paenibacillus albidus* | R32 b 2012 | 17 | 0 | 1 | 0 | 0,3 | 1 | 1 | 1 | 1 |
| *Paenibacillus albidus* | R32b 2014 | 18 | 0 | 1 | 0 | 0,3 | 1 | 1 | 1 | 1 |
| *Paenibacillus pabuli* | A14b | 7 | 0 | 1 | 0 | 0,3 | 1 | 1 | 1 | 1 |
| *Paenibacillus pabuli* | A15b | 8 | 0 | 1 | 0 | 0,3 | 1 | 1 | 1 | 1 |
| *Paenibacillus taichungensis* | E40b | 13 | 0 | 1 | 0 | 0,3 | 1 | 1 | 1 | 1 |
| *Paenibacillus xylanexendes* | B23b | 9 | 0 | 1 | 0 | 0,3 | 1 | 1 | 1 | 1 |
| *Pediococcus pentosaceus* | 20336 | 23 | 0 | 1 | 0 | 0,3 | 2 | 2 | 2 | 2 |
| *Pediococcus pentosaceus* | ATCC 744 | 24 | 0 | 1 | 0 | 0,3 | 2 | 2 | 2 | 2 |
| *Pediococcus pentosaceus* | LMG 11488 | 25 | 0 | 1 | 1 | 0,8 | 2 | 2 | 2 | 2 |
| *Pantoea agglomerans* | LMG 2565 | LMG 2565 | 0 | 0 | 0 | 0 | 0 | 0 | 0 | 0 |
| *Pantoea agglomerans* | MR2-2 | MR2-2 | 2 | 2 | 2 | 2 | 0 | 0 | 0 | 0 |
| *Pantoea agglomerans* | RP4 | RP4 | 0 | 0 | 0 | 0 | 0 | 0 | 0 | 0 |
| *Pantoea agglomerans* | RR1-2 | RR1-2 | 2 | 1 | 0 | 0,7 | 0 | 0 | 0 | 0 |
| *Pantoea agglomerans* | RR2-2 | RR2-2 | 3 | 3 | 2 | 2,5 | 0 | 0 | 0 | 0 |
| *Pseudomonas alcaliphila* | MP1 | MP1 | 0 | 0 | 0 | 0 | 0 | 0 | 0 | 0 |
| *Pectobacterium carotovorum actinidiae* | 26003 | 26003 | 0 | 0 | 0 | 0 | 0 | 0 | 0 | 0 |
| *Pectobacterium carotovorum carotovorum* | LMG 2404 | LMG 2404 | 1 | 0 | 0 | 0,2 | 0 | 0 | 0 | 0 |
| *Pseudomonas chicorii* | I3C | I3C | 1 | 0 | 0 | 0,2 | 0 | 0 | 0 | 0 |
| *Pseudomonas fluorescens* | L1A | L1A | 1 | 1 | 0 | 0,5 | 0 | 0 | 0 | 0 |
| *Pseudomonas fragi* | PS25 | PS25 | 2 | 1 | 1 | 1,2 | 0 | 0 | 0 | 0 |
| *Pseudomonas gessardi* | LMG 21604 | LMG 21604 | 2 | 2 | 0 | 1 | 0 | 0 | 0 | 0 |
| *Pseudomonas gessardi* | Pz20 | Pz20 | 0 | 0 | 0 | 0 | 0 | 0 | 0 | 0 |
| *Pseudomonas gessardi* | Pz20 | Pz20 | 1 | 0 | 0 | 0,2 | 0 | 0 | 0 | 0 |
| *Pseudomonas koreensis* | MP2 | MP2 | 2 | 2 | 1 | 1,5 | 0 | 0 | 0 | 0 |
| *Pseudomonas koreensis* | RR2-1 | RR2-1 | 0 | 0 | 0 | 0 | 0 | 0 | 0 | 0 |
| *Pseudomonas koreensis* | A3 | A3 | 0 | 0 | 0 | 0 | 0 | 0 | 0 | 0 |
| *Pseudomonas lundensis* | 25E | 25E | 1 | 0 | 0 | 0,2 | 0 | 0 | 0 | 0 |
| *Pseudomonas luteola* |  |  | 0 | 0 | 0 | 0 | 0 | 0 | 0 | 0 |
| *Pseudomonas luteola* | LR2-2 | LR2-2 | 2 | 2 | 1 | 1,5 | 0 | 0 | 0 | 0 |
| *Pseudomonas marginalis* | LMG 2210 | LMG 2210 | 0 | 0 | 0 | 0 | 0 | 0 | 0 | 0 |
| *Pseudomonas marinicola* | A2 | A2 | 0 | 0 | 0 | 0 | 0 | 0 | 0 | 0 |
| *Pseudomonas monteilii* | RP1 | RP1 | 3 | 3 | 3 | 3 | 0 | 0 | 0 | 0 |
| *Pseudomonas oleovorans* | RR1-1 | RR1-1 | 2 | 2 | 2 | 2 | 0 | 0 | 0 | 0 |
| *Pseudomonas putida* | MR2-1 | MR2-1 | 1 | 0 | 0 | 0,2 | 0 | 0 | 0 | 0 |
| *Pseudomonas putida* | A1 | A1 | 0 | 0 | 0 | 0 | 0 | 0 | 0 | 0 |
| *Pseudomonas putida* | I1B | I1B | 0 | 0 | 0 | 0 | 0 | 0 | 0 | 0 |
| *Pseudomonas rodhesise* | RP3 | RP3 | 1 | 0 | 0 | 0,2 | 0 | 0 | 0 | 0 |
| *Pseudomonas rodhesise* | LR1-1 | LR1-1 | 0 | 0 | 0 | 0 | 0 | 0 | 0 | 0 |
| *Pseudomonas taetrolens* | PS3 | PS3 | 2 | 1 | 0 | 0,7 | 0 | 0 | 0 | 0 |
| *Pseudomonas taetrolens* | PS4 | PS4 | 1 | 0 | 0 | 0,2 | 0 | 0 | 0 | 0 |
| *Pseudomonas yamanorum* | PS5 | PS5 | 1 | 1 | 0 | 0,5 | 0 | 0 | 0 | 0 |
| *Pseudomonas endophtytica* | PS2 | PS2 | 1 | 1 | 1 | 1 | 0 | 0 | 0 | 0 |
| *Pseudomonas extremalis* | C4 | C4 | 0 | 0 | 0 | 0 | 0 | 0 | 0 | 0 |
| *Pseudomonas fluorescens* | 17298 | 17298 | 2 | 1 | 1 | 1,2 | 0 | 0 | 0 | 0 |
| *Pseudomonas fluorescens* | A18 | A18 | 0 | 0 | 0 | 0 | 0 | 0 | 0 | 0 |
| *Pseudomonas paralactis* | B1 | B1 | 0 | 0 | 0 | 0 | 0 | 0 | 0 | 0 |
| *Pseudomonas putida* | PB33 | PB33 | 0 | 0 | 0 | 0 | 0 | 0 | 0 | 0 |
| *Pseudomonas spp* | MT9 | MT9 | 0 | 0 | 0 | 0 | 0 | 0 | 0 | 0 |
| *Pseudomonas spp* | T0BU1 | T0BU1 | 0 | 0 | 0 | 0 | 0 | 0 | 0 | 0 |
| *Pseudomonas spp* | T0BU2 | T0BU2 | 0 | 0 | 0 | 0 | 0 | 0 | 0 | 0 |
| *Pseudomonas spp* | T16P20 | T16P20 | 1 | 0 | 0 | 0,2 | 0 | 0 | 0 | 0 |
| *Pseudomonas spp* | MT9 | MT9 | 0 | 0 | 0 | 0 | 0 | 0 | 0 | 0 |
| *Pseudomonas taetrolens* | PS1 | PS1 | 2 | 2 | 1 | 1,5 | 0 | 0 | 0 | 0 |
| *Pseudomonas weihenstephanensis* | A15 | A15 | 1 | 0 | 0 | 0,2 | 0 | 0 | 0 | 0 |
| *Pseudomonas weihenstephanensis* | T16BU9 | T16BU9 | 1 | 0 | 0 | 0,2 | 0 | 0 | 0 | 0 |
| *Serratia liquefaciens* | A17 | A17 | 3 | 3 | 3 | 3 | 0 | 0 | 0 | 0 |
| *Serratia liquefaciens* | A19 | A19 | 1 | 0 | 0 | 0,2 | 0 | 0 | 0 | 0 |
| *Serratia marcescens* | 8T11 | 8T11 | 3 | 3 | 3 | 3 | 0 | 0 | 0 | 0 |
| *Sphingomonas spp.* | SVA | 21 | 1 | 2 | 0 | 0,8 | 1 | 1 | 1 | 1 |
| *Staphylococcus pasterii* | SB26 | 22 | 1 | 2 | 2 | 1,8 | 1 | 2 | 2 | 1,8 |

**Table S2.** Yeast strains screened against red and yellow onion skins-based media. The Reference code is an internal code used to univocally identify the strain used in this study.

| Genus or Species | Strain | Reference  code | Red onion skins | | | | Yellow onion skins | | | |
| --- | --- | --- | --- | --- | --- | --- | --- | --- | --- | --- |
|  |  |  | **15%** | **20%** | **25%** | **WMG** | **15%** | **20%** | **25%** | **WMG** |
| *Aeurobasidium pullulans* | YLq1 | 58 | 2 | 2 | 3 | 2,5 | 2 | 2 | 2 | 2 |
| *Candida boidinii* | A5y | 33 | 1 | 2 | 1 | 1,3 | 3 | 3 | 3 | 3 |
| *Candida boidinii* | E52y | 34 | 1 | 2 | 1 | 1,3 | 3 | 3 | 3 | 3 |
| *Candida boidinii* | F28y | 35 | 1 | 2 | 1 | 1,3 | 3 | 3 | 3 | 3 |
| *Candida parapsilosis* | MUCL 31233 | 36 | 2 | 3 | 2 | 2,3 | 3 | 3 | 3 | 3 |
| *Candida parapsilosis* | YB51 | 59 | 2 | 2 | 3 | 2,5 | 2 | 2 | 2 | 2 |
| *Candida tropicalis* | 1M1 | 37 | 2 | 2 | 2 | 2 | 3 | 3 | 3 | 3 |
| *Candida zemplinina* |  | 38 | 1 | 1 | 1 | 1 | 3 | 2 | 1 | 1,7 |
| *Debaryomyces hansenii* | BC T3-23 | 70 | 2 | 2 | 2 | 2 | 2 | 2 | 2 | 2 |
| *Geotrichum candidum* | 1C1 | 39 | 1 | 2 | 1 | 1,3 | 3 | 2 | 1 | 1,7 |
| *Geotrichum candidum* | 1G1 | 40 | 1 | 2 | 1 | 1,3 | 3 | 2 | 1 | 1,7 |
| *Geotrichum candidum* | 4M1 | 41 | 1 | 2 | 1 | 1,3 | 3 | 2 | 1 | 1,7 |
| *Geotrichum candidum* | 11G1 | 42 | 1 | 2 | 1 | 1,3 | 2 | 2 | 2 | 2 |
| *Geotrichum candidum* | 14G1 | 43 | 2 | 2 | 1 | 1,5 | 2 | 2 | 2 | 2 |
| *Hanseniaspora uvarum* | 8795 | 60 | 2 | 2 | 1 | 1,5 | 2 | 2 | 2 | 2 |
| *Hanseniaspora uvarum* | 8801 | 61 | 2 | 2 | 1 | 1,5 | 2 | 2 | 2 | 2 |
| *Hanseniaspora uvarum* | 8802 | 62 | 2 | 2 | 1 | 1,5 | 2 | 2 | 2 | 2 |
| *Hanseniaspora uvarum* | 8807 | 63 | 2 | 2 | 1 | 1,5 | 2 | 2 | 2 | 2 |
| *Hanseniaspora uvarum* |  | 64 | 2 | 2 | 1 | 1,5 | 2 | 2 | 2 | 2 |
| *Hanseniaspora uvarum* |  | 65 | 2 | 2 | 1 | 1,5 | 2 | 2 | 2 | 2 |
| *Kluyveromyces marxianus* |  | 44 | 2 | 2 | 1 | 1,5 | 2 | 2 | 2 | 2 |
| *Metschnikowia pulcherrima* |  | 45 | 2 | 3 | 1 | 1,8 | 2 | 2 | 2 | 2 |
| *Metschnikowia pulcherrima* | Y1D | 66 | 2 | 2 | 1 | 1,5 | 2 | 2 | 2 | 2 |
| *Pichia anomala* | MUCL 28639 | 46 | 1 | 3 | 2 | 2,1 | 2 | 2 | 2 | 2 |
| *Pichia fermentans* | 9C1 | 49 | 1 | 2 | 1 | 1,3 | 2 | 2 | 2 | 2 |
| *Pichia holstii* | 8G | 50 | 1 | 2 | 1 | 1,3 | 2 | 2 | 2 | 2 |
| *Pichia holstii* | 13A | 51 | 0 | 2 | 1 | 1,1 | 2 | 2 | 2 | 2 |
| *Pichia manshurica* |  | 47 | 1 | 2 | 2 | 1,8 | 2 | 2 | 2 | 2 |
| *Pichia membranifaciens* | MUCL 29895 | 48 | 1 | 2 | 1 | 1,3 | 2 | 2 | 2 | 2 |
| *Pichia membranifaciens* | 6C | 52 | 2 | 2 | 2 | 2 | 2 | 2 | 2 | 2 |
| *Pichia norvegensis* | COR2y | 67 | 2 | 2 | 1 | 1,5 | 2 | 2 | 2 | 2 |
| *Rhodotorula diobovata* | YB1 | 68 | 3 | 3 | 3 | 3 | 2 | 2 | 2 | 2 |
| *Rhodotorula mucilaginosa* | MUCL 30403 | 53 | 2 | 2 | 1 | 1,5 | 2 | 2 | 2 | 2 |
| *Rhodotorula mucilaginosa* |  | 69 | 1 | 1 | 1 | 1 | 2 | 2 | 2 | 2 |
| *Saccharomyces cerevisiae* | 8C | 54 | 1 | 2 | 1 | 1,3 | 2 | 2 | 2 | 2 |
| *Saccharomyces cerevisiae* | 10A | 55 | 2 | 2 | 1 | 1,5 | 3 | 4 | 3 | 3,3 |
| *Saccharomyces cerevisiae* | ENARTIS SC (EN SC) | 71 | 1 | 1 | 2 | 1,5 | 3 | 3 | 4 | 3,5 |
| *Saccharomyces cerevisiae* | LALVIN RHONE 2056 | 72 | 1 | 1 | 2 | 1,5 | 2 | 2 | 2 | 2 |
| *Saccharomyces cerevisiae Wine* | LALVIN BM45 | 73 | 1 | 1 | 1 | 1 | 2 | 2 | 2 | 2 |
| *Saccharomyces cerevisiae* | LI 60-17 | 74 | 1 | 1 | 1 | 1 | 2 | 2 | 2 | 2 |
| *Saccharomyces cerevisiae* | LI 180-7 | 75 | 1 | 1 | 1 | 1 | 2 | 2 | 2 | 2 |
| *Saccharomyces cerevisiae* | L P F | 76 | 1 | 1 | 1 | 1 | 2 | 2 | 2 | 2 |
| *Saccharomyces cerevisiae* | KI 30-1 | 77 | 2 | 2 | 2 | 2 | 2 | 2 | 2 | 2 |
| *Saccharomyces cerevisiae* | LALVIN D80 | 78 | 2 | 2 | 2 | 2 | 2 | 2 | 2 | 2 |
| *Trichosporon mycotoxinivorans* |  | 56 | 2 | 3 | 2 | 2,3 | 2 | 2 | 2 | 2 |
| *Zygosaccharomyces mrakii* | CL 30-29 | 57 | 2 | 3 | 3 | 2,8 | 2 | 2 | 2 | 2 |
